# A liquid biopsy-RNAseq method for monitoring the expression of genes involved in drug disposition: Proof-of-concept application to cholestatic liver disease

**DOI:** 10.1016/j.jpba.2025.117244

**Published:** 2025-11-10

**Authors:** Amit Dahal, Teresa Sierra, Colleen M. Hayes, David N. Assis, Amin Rostami-Hodjegan, Nisanne S. Ghonem, Brahim Achour

**Affiliations:** a Department of Biomedical and Pharmaceutical Sciences, College of Pharmacy, University of Rhode Island, Kingston, RI, USA; b Section of Digestive Diseases, Yale School of Medicine, New Haven, CT, USA; c Centre for Applied Pharmacokinetic Research, University of Manchester, Manchester, UK; d Certara Predictive Technologies, Sheffield, UK

**Keywords:** Liquid biopsy, Extracellular vesicles, RNAseq, Physiologically based pharmacokinetics, Cholestatic liver disease

## Abstract

Between-patient variability in metabolism and disposition of therapeutic drugs is associated with variable expression and activity of enzymes and transporters. Direct measurement of the expression of such proteins in relevant organs is limited by access to tissue biopsies. Plasma-derived extracellular vesicles (EVs), sampled as liquid biopsy, offer a minimally invasive and widely accessible alternative to tissue biopsy. This report provides a technical account for the extraction, characterization and RNAseq of EVs from plasma from healthy donors and donors with cholestatic liver disease (*n* = 3 in each group). Quality control (QC) steps implemented at different stages of the workflow ensured quality of the EVs and extracted RNA for downstream analysis. These QCs included visualization, size distribution, protein content and RNA quality and yield. Extracted EVs were a mixture of small and large vesicles (35–350 nm). Replicate RNAseq data reflected strong correlation (Pearson’s *r* = 1, *p* < 0.001), low bias (average fold error = 1) and limited scatter (absolute average fold error = 1.34) across replicates. The method enabled monitoring RNA expression of 656 pharmacologically relevant genes, including 199 xenobiotic-metabolizing enzymes, 444 transporters, and 11 transcription factors. Disease perturbation in expression from healthy baseline was estimated for 93 % of monitored pharmacokinetic pathways. The data were used, in combination with demographic and serum albumin changes, to generate a cholestatic liver disease pharmacokinetic model. The model reflected exposure to budesonide and cyclosporine consistent with early-stage disease. The model also reflected increased total serum bilirubin, likely due to dysregulation of hepatobiliary transport.

## 1. Introduction

Inter-individual variability in drug response presents a major challenge for healthcare providers, often leading to adverse drug reactions or suboptimal clinical outcomes, which necessitate treatment cessation or a suitable dosing adjustment [1]. Initiatives to improve on the current “one-size-fits-all” approach to pharmacotherapy require defining key patient characteristics that determine the fate of drugs in the body [2]. Currently implemented patient characterization methods typically generate genotype or phenotype data related to key pharmacokinetic (PK) pathways. Genotyping of polymorphic genes involved in drug disposition (e.g., CYP2D6 [3]) offers a relatively non-quantitative indication of functional activity, while phenotyping with exogenous probes (such as the ‘Geneva cocktail’ [4]) requires oral administration of drugs to patients and dedicated bioanalytic settings with inherent logistical challenges. Whereas endogenous biomarkers of metabolism and transport capacity (e.g., serum 4β-hydroxycholesterol-to-cholesterol ratio for CYP3A activity [5] and serum coproporphyrin I for OATP1B1 activity [6]) have the advantage of being non-invasive, they often lack selectivity. Robust liquid biopsy assays have therefore been proposed recently to complement, and in some cases, replace such approaches, with the caveat that the technique should be able to define a quantitative grade as a proxy for a patient’s individual drug disposition capacity [2, 7].

Liquid biopsy involves sampling of biofluids, such as plasma/serum or urine, extraction of extracellular vesicles (EVs), and analysis of their biomolecular composition using ‘omics’ techniques to monitor therapeutic and diagnostic biomarkers of disease [8-12]. As a general approach, EVs from patients and healthy controls are compared to establish deviation from baseline with respect to expression levels, degrees of variability and/or polymorphic profiles of the genes/proteins that mediate pathways relevant to disease development or pharmacologic response [11]. We previously reported a novel liquid biopsy assay and demonstrated its utility in assessing *in vivo* activity of pathways relevant to drug exposure in patients with cardiovascular disease [9] and chronic kidney disease [13]. However, the methodological workflow of the assay and technical considerations in relation to its implementation have not been reported. We therefore describe, in this report, the deployment of a liquid biopsy-RNAseq strategy with quality control steps at the stages of EV extraction, EV characterization, and RNA sequencing, followed by a proof-of-concept application of the approach with physiologically based pharmacokinetic (PBPK) modelling using a small set of plasma samples from healthy donors and donors with cholestatic liver disease.

## 2. Materials and methods

**Fig. 1.**
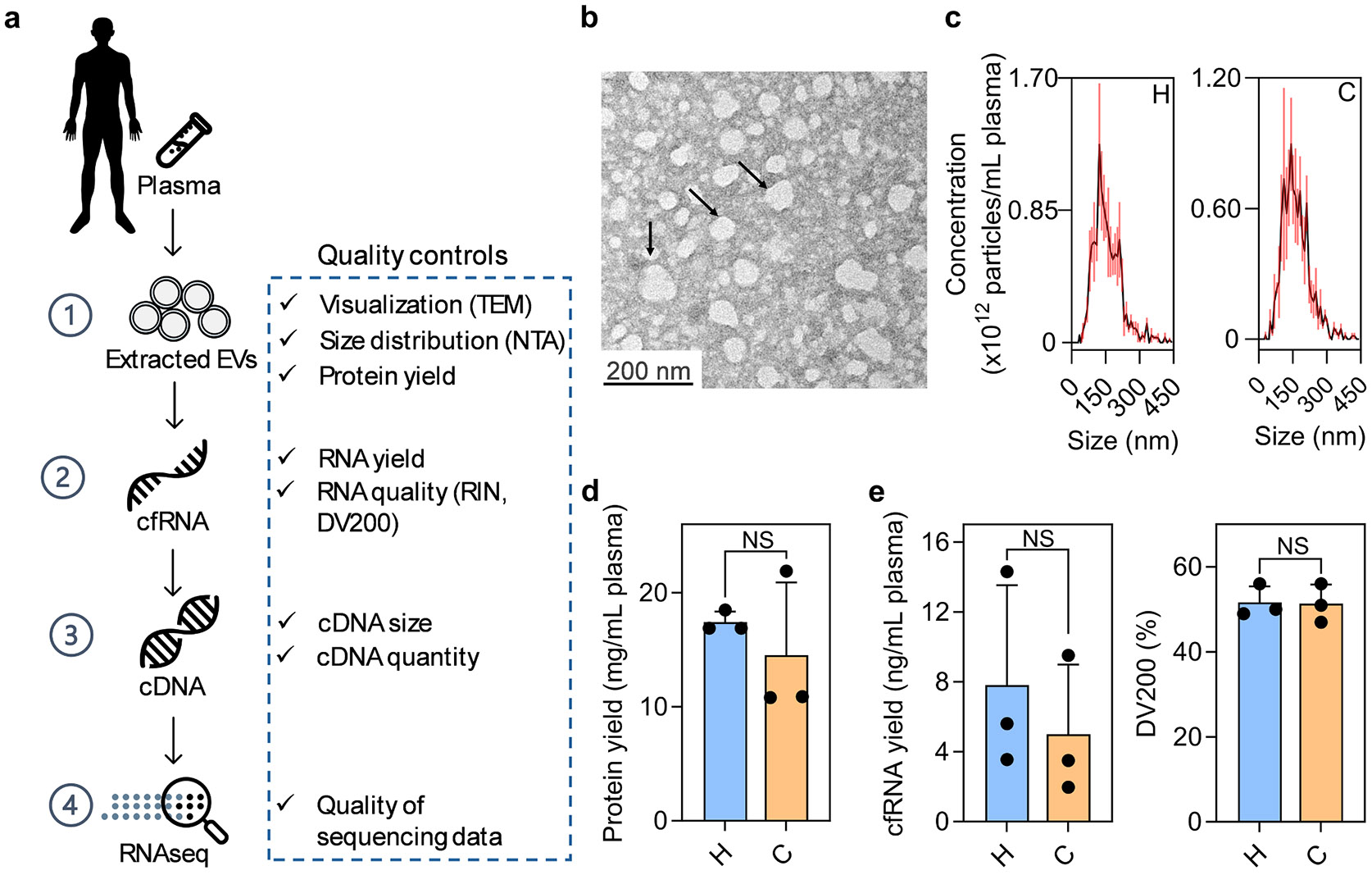
Experimental workflow and quality controls of isolated EVs. The experimental protocol includes extraction of plasma EVs, isolation of cfRNA from EVs, reverse transcription to cDNA from cfRNA, preparation of cDNA libraries, and finally RNAseq (a). Quality controls of EVs include visualization with transmission electron microscopy (b), size distribution characterization (c), assessment of EV protein content (d), and total cfRNA yield and quality (e). Size distribution reflected EV size range of 35–300 nm, with a number-weighted mean size of 152 and 162 nm in plasma from healthy controls and donors with cholestatic liver disease, respectively. Protein yield above 10 mg/mL plasma is considered acceptable. Yield of cfRNA is normally > 1 ng/mL plasma, and DV200 > 30 % is required for good quality sequencing (Fig S2). cDNA target size is typically approximately 260–300 bp with high quantity from each sample to prepare cDNA libraries (Fig S3). Sequencing should be of sufficient depth (> 30 M reads/sample) and quality (>75 % of bases of Q30 or higher scores) (Fig. 2). In panel c, size distribution data are plotted as mean and standard error of the mean. In panels d and e, protein yield and RNA yield and quality data are plotted as mean and SD. Abbreviations: C, cholestatic liver disease; cDNA, complementary DNA; cfRNA, cell-free RNA; DV200, the percentage of RNA fragments longer than 200 nucleotides; EV, extracellular vesicle; H, healthy; NTA, nanoparticle tracking analysis; RIN, RNA integrity number; RNAseq, RNA sequencing; TEM, transmission electron microscopy; NS, non-significant differences based on an unpaired *t*-test.

The experimental workflow is summarized in Fig. 1a. The protocol is presented in Supplementary Information, with notes, warnings and quality control (QC) measures. QC steps are listed in Table S1.

### 2.1. Human plasma samples

Plasma samples from donors with cholestatic liver disease, including primary sclerosing cholangitis (PSC, n = 1, male) and primary biliary cholangitis (PBC, n = 2, female), age range 62–72 years, were provided by the Yale University Liver Center Clinical Registry (New Haven, CT, USA). All donors provided written consent to have their blood samples collected, de-identified and stored at the Yale University Liver Center Clinical Registry. The de-identified samples were determined to be exempt from the Institutional Review Board at Yale University and the University of Rhode Island (Kingston, RI). The study protocol conformed to the ethical guidelines of the 1975 Declaration of Helsinki, as reflected in prior approval by the institutional review committee. Plasma from healthy de-identified adult donors (BioIVT, Westbury, NY, USA) served as controls (3 female; age range 35–55 years). Donor demographic and clinical information is summarized in Table 1. Pooled plasma-derived cell-free RNA (cfRNA) from three healthy donors (1 female, 2 male; age range: 23–57 years) (BioIVT, West Sussex, UK) was used to assess the reproducibility of the EV-RNAseq method.

### 2.2. Extraction of extracellular vesicles from plasma

EVs were extracted from platelet-depleted plasma samples (1 mL each) using polymer-assisted precipitation, as previously reported [12]. Polymer-assisted precipitation was performed using ExoQuick reagent (System Biosciences, Palo Alto, CA, USA), following the manufacturer’s instructions, with some modifications. Briefly, plasma samples were thawed on ice, mixed thoroughly and centrifugated at 3000 *g* for 15 min at 4° C to remove cell debris. An aliquot of 1 mL of plasma supernatant was diluted with 0.5 mL sterile, nuclease-free 1X PBS, pH 7.4. EV precipitation polymer (ExoQuick reagent) was added to the plasma samples at 1:4 vol ratio, followed by thorough mixing by inverting the tubes. The mixtures were incubated (60 min, 4°C) and EVs were collected by gentle centrifugation (1500 *g*, 45 min, 4°C). Precipitated EVs appeared as a beige/white pellet (Fig S1). Each pellet underwent three wash cycles with 100 μL of 1X PBS, pH 7.4, followed by centrifugation at 1500 *g* for 5 min at 4°C after each wash step. Supernatants were discarded, and the pellets were reconstituted in 200 μL of nuclease-free 1X PBS, pH 7.4.

### 2.3. Transmission electron microscopy

Plasma-derived EVs were prepared for transmission electron microscopy by applying 5 μL of suspended EVs directly onto the surface of 200-mesh carbon-coated copper grids. Grids were incubated for 5 min at room temperature to allow adsorption, followed by gentle blotting to remove excess liquid. The grids were then air-dried for 10 min at room temperature. Fixation was performed by immersing the grids in a drop of 2.5 % glutaraldehyde in sodium cacodylate buffer for 2 min at room temperature. Subsequently, the grids were washed through five sequential drops of autoclaved ultrapure water, each for 30 s. Negative staining was conducted by placing the grids in a drop of 2 % aqueous uranyl acetate for 2 min at room temperature. After staining, the excess solution was carefully blotted, and the grids were air-dried on filter paper for 15 min prior to imaging. EVs were visualized by transmission electron microscopy performed on a JEOL F200 electron microscope (Peabody, MA, USA) operating at 200 kV, equipped with a cold field emission gun and a Gatan Continuum S electron energy loss spectrometer (EELS). Images were captured using a Gatan Rio9 CMOS camera at various magnifications. Image processing was done using ImageJ software version 1.54 g.

### 2.4. Nanoparticle tracking analysis

Size distribution and concentration of EVs were measured using ZetaView MONO (Particle Metrix, Inning am Ammersee, Germany). The instrument was calibrated using 100 nm Nanosphere Standards (Applied Microspheres, Utrecht, Netherlands) to ensure accurate settings. EVs were diluted in sterile, filtered PBS, pH 7.2 (Gibco, Thermo Fisher Scientific, Waltham, MA, USA). To obtain precise measurements, optimal measurement concentrations were determined by pre-testing to achieve particle-per-frame values (50–400 particles/frame) within the manufacturer-recommended range. Nanoparticle tracking analysis was performed using the built-in ZetaView Software 8.05.16 SP7 with the manufacturer’s default settings. All samples were analyzed in triplicate. Images were taken at 11 different positions for each replicate and averaged to determine concentrations and particle size measurements.

### 2.5. Measurement of total protein content of plasma EVs

Total protein content of EV samples was measured using a bicin-choninic acid protein assay kit (EMD Millipore, Billerica, MA, USA), following the manufacturer’s instructions. Bovine serum albumin was used as a calibration standard. All measurements were performed in triplicate.

### 2.6. Sample preparation of EVs for proteomics

To assess the EVs for plasma contaminants and generic membrane protein markers, protein from individual EV samples was processed for proteomic analysis using an optimized filter-aided sample preparation protocol, as previously described [14]. Briefly, 50 μg of EV protein from each sample was solubilized and reduced with sodium deoxycholate (10 % w/v final concentration) and 1,4-dithiothreitol (20 mM final concentration) by incubation for 30 min at 56°C. Amicon Ultra 0.5 mL centrifugal filter units with a 10-kDa molecular weight cutoff (Sigma-Aldrich, Burlington, MA, USA) were preconditioned with two 200 μL volumes of 0.1 M Tris-HCl, pH 8.5. Protein samples were then transferred to the filters and centrifuged at 13,000 rpm for 20 min at room temperature. This was followed by two wash steps with 200 μL of 8 M urea in 0.1 M Tris-HCl, pH 8.5, and cysteine alkylation with 100 μL of 50 mM iodoacetamide (with 8 M urea in 0.1 M Tris-HCl, pH 8.5) in the dark for 30 min. Two wash steps were carried out with 200 μL of 8 M urea in 0.1 M Tris-HCl (pH 8.5), followed by buffer exchange with 200 μL of 1 M urea in 50 mM ammonium bicarbonate (pH 8.0). Proteolytic digestion was carried out with lysyl endopeptidase (enzyme-to-protein ratio 1:50, 3 h, 30°C), followed by trypsin (enzyme-to-protein ratio 1:25, 13 h, 37°C). The peptides were collected by centrifugation (14,000 rpm, 20 min, room temperature), followed by two additional elutions with 100 μL of 0.5 M sodium chloride, centrifuged under the same conditions. The peptides were desalted using Pierce C18 spin columns (Thermo Fisher Scientific, Waltham, MA, USA), following the manufacturer’s protocol. The desalted peptides were lyophilized to dryness and stored at −80°C until subsequent proteomics analysis.

### 2.7. EV proteomics with liquid chromatography-tandem mass spectrometry

Peptides were trapped and eluted from a PepMap Neo (300 μm x 5 mm) trap column using a Vanquish Neo UHPLC nano system (Thermo Scientific, Waltham, MA, USA), which kept the samples at 11°C before injection. Peptide mixtures were separated on a reversed-phase Ion-Opticks-TS analytical column (25 cm×75 μm, 1.7 μm particle size C18 resin) supported by an EASY-Spray nano-source and stabilized with a Heater THOR Controller (Ion-Opticks) at 55°C. Peptides were eluted at a flow rate of 0.35 μL/min using a 35-min gradient, from 98 % buffer A and 2 % buffer B to 5.5 % buffer B at 0.1 min, to 44 % buffer B at 27.1 min, followed by a column wash with 55 % buffer B at 29.7 min, to 99 % buffer B at 35 min, and finally, followed by equilibration back to 98 % buffer A and 2 % buffer B. Buffer A consisted of 0.1 % formic acid and 0.5 % acetonitrile in HPLC-grade water. Buffer B consisted of 80 % acetonitrile, 20 % HPLC-grade water and 0.1 % formic acid. Eluted peptides were ionized by electrospray (2.5 kV), followed by mass spectrometric analysis on an Orbitrap Astral mass spectrometer (Thermo Scientific). Precursor spectra were acquired with the following parameters: *m/z* range of 380–980 Th, 240,000 resolution, normalized AGC target of 200 %, and maximum injection time of 3 ms. Data-independent acquisition (DIA) on the mass spectrometer was configured to acquire 149, 4-Th windows from 380 to 980 Th, with 25 % HCD collision energy, normalized AGC target of 100 %, and maximum injection time of 3 ms.

Following acquisition, data were searched using Spectronaut version 19.5 (Biognosys, Newton, MA, USA) against UniProt *Homo sapiens* database (Proteome ID: UP000005640, 1st version of 2025) using the directDIA method with an identification precursor and protein q-value cutoff of 1 %, ‘generate decoys’ set to true, the protein inference workflow set to maxLFQ, inference algorithm set to IDPicker, quantity level set to MS2, cross-run normalization set to false, and the protein grouping quantification set to median peptide and precursor quantity. The total protein approach (TPA) was used for quantification, based on the ratio of the summed MS signal intensity assigned to one protein relative to the total MS signal intensity of the sample, converted to units of pmol/mg total EV protein using the molecular mass of the target protein, as reported previously [15,16].

### 2.8. Extraction and characterization of cell-free RNA from EVs

Total cfRNA was extracted from plasma-derived EVs using the MagMax Cell-Free Total Nucleic Acid Isolation Kit (Thermo Fisher Scientific, Austin, TX, USA), following the manufacturer’s instructions. A detailed protocol is included in Supplementary Information. The quantity and quality of cfRNA were assessed using the Bioanalyzer 2100 system (Agilent, Santa Clara, CA, USA) and the RNA 6000 Pico Kit, following the manufacturer’s protocol. RNA yield, reported as total cfRNA per mL of plasma, and RNA quality, evaluated using the DV200 score (the percentage of RNA fragments longer than 200 nucleotides), were determined using 2100 Expert Software version B.02.12 2420 (Agilent) (Fig S2).

### 2.9. Preparation of cfRNA for sequencing

Total cfRNA extracted from plasma-derived EVs was prepared for RNAseq. Reverse transcription was performed with 3.5 μL of each cfRNA sample using AmpliSeq cDNA Synthesis for Illumina (San Diego, CA, USA). The complementary DNA (cDNA, 5 μL) was used in target amplification by polymerase chain reaction (PCR, 16 cycles) using AmpliSeq Transcriptome Human Gene Expression Panel and AmpliSeq HiFi Mix (Illumina). Libraries were prepared for sequencing using AmpliSeq Library PLUS (96 reactions). Amplicon libraries were further amplified (7 cycles). Library sizes were assessed using TapeStation 4200 (Agilent). The library sizes ranged from 280 to 299 bp, with an average library size of 287 bp (Fig S3). Each library was quantitated by quantitative PCR (qPCR) with a Roche LightCycler96 using FastStart Essential Green Master (Roche) and KAPA Library Quant (Illumina) DNA standards (KAPA KK4903). Universal primers complementary to adapter flow cell binding sites (P5 and P7) were used. Normalized libraries (2 nM) were pooled. Two aliquots of the pooled libraries were used to target 130 pM loading concentration (Lane 1) and 140 pM loading concentration (Lane 2), following 0.2 N NaOH denaturation of cDNA and spike-in with 5 % PhiX control library. On-board clustering and sequencing were performed on an Illumina NovaSeq X Plus at 150-cycle paired-end resolution. Bcl2fastq conversion was completed using on-board DRAGEN software (Illumina).

### 2.10. Analysis of RNAseq data

The AmpliSeq panel targets approximately 20,800 human RefSeq genes with *>* 18,300 protein-coding genes. The read quality was assessed using FastQC software [17]. RNA-sequencing data QC did not reveal the presence of adapters that required trimming. The reads were mapped to the human genome (GRCh38) using STAR software version 2.7.11b [18]. Transcript abundance was measured for the genes in the panel (the list of 20,800 genes in the panel was provided by the vendor) using featureCounts software [19]. Expression normalization and differential gene expression calculations were performed using DESeq2 software to identify statistically significant differentially expressed genes [20]. Liver-specific markers (used to describe shedding) were selected based on previously defined criteria [12], with the exclusion of genes coding for blood clotting factors (F2, F9 and FGB) to avoid conflating shedding with disease-driven changes in expression anticipated in liver disease [21]. Monitored targets included drug and xenobiotic-metabolizing enzymes (XMEs), ATP-binding cassette (ABC) transporters, solute carriers (SLCs), FcRn, and transcription factors that regulate the expression of enzymes and transporters (listed in Table S2). Expression levels were recorded relative to the total number of reads in each sample as reads per million (RPM).

### 2.11. Statistical analysis and data annotation

Statistical analysis was carried out using Microsoft Excel 2016 and GraphPad Prism version 10.0.2 (GraphPad Software, La Jolla, CA, USA). Heatmap generation and hierarchical cluster analysis (HCA) were carried out using Morpheus software (Broad Institute, Cambridge, MA, USA, https://software.broadinstitute.org/morpheus/). Graphs were generated using GraphPad Prism and Microsoft PowerPoint. Expression data are presented as mean and standard deviation (SD). Data describing protein yield and RNA quality and yield are presented as mean and SD. Size distribution data are presented as mean and standard error of the mean. Replicate analysis was carried out using Pearson correlation, linear regression, average fold error (AFE, Eq. 1) and absolute average fold error (AAFE, Eq. 2), where $x_{i}$ and $y_{i}$ are replicate measurements of the same gene i.


(1) [∑i=1nlog(xi∕yi)n]AFE=10

(2) [∑i=1n∣lor(xi∕yi)∣n]AAFE=10

For HCA, heat-mapped, natural log-transformed data (*n* = 20,725 genes) were clustered by Euclidean distance to align samples (columns) with similar expression levels. Data (*n* = 20,725 genes) were also analyzed using principal components analysis (PCA) and plotted with percent variance components using ClustVis webtool [22]. Volcano plots used the following criteria: data were compared strictly when a quantitative expression above the limit of quantification was available in all samples (*n* = 18,771 genes), a cutoff fold change of at least 3-fold was considered, and *p* < 0.05 (unpaired *t*-test) was used for statistical significance. Enriched or suppressed genes were annotated using Gene Ontology classification (PANTHER database, http://www.pantherdb.org/). The selected targets (enzymes, transporters and transcription factors) were assessed for disease effects using an unpaired *t*-test, with a cutoff *p*-value of 0.05. The Human Protein Atlas (https://www.proteinatlas.org/) (Version 24.0, updated on 10–22–2024) [23] was used to assess tissue-specific enrichment of gene expression, and Ingenuity Pathway Analysis (QIAGEN, https://www.qiagenbioinformatics.com/products/ingenuitypathway-analysis) was used to annotate data for location of coded proteins and druggable pathways.

### 2.12. Physiologically based pharmacokinetic modelling (PBPK) in cholestatic liver disease

A cholestatic liver disease population model was developed using the default Sim-Healthy Volunteers Population model available on the Simcyp Simulator v24 (Certara Predictive Technologies, Sheffield, UK) as a base model. The model was adapted by changing the demographic distribution, serum albumin levels and expression of key drug disposition pathways to reflect changes in PBC. PBC has higher incidence in women (female to male ratio 9:1), and serum albumin levels are reported to decrease (≤4 g/dL) over the course of disease progression [24]. Hepatic blood flow was not changed, and intestinal and renal functions were assumed to be unaffected. Scaling of intrinsic metabolic and transporter clearance was based on abundance fold change (FC), expressed as ratios of mean disease expression relative to mean healthy expression of hepatic drug-metabolizing enzymes (cytochrome P450s/CYPs and glucuronosyltransferases/UGTs) and drug transporters (OATP1B1, MRP2/3, P-gp, BSEP, BCRP) using RNAseq data (Eq. 3), assuming linear correlation between cfRNA, protein and functional activity, as previously investigated in small sample sets [9,10,13,25]. Expression values were considered when data were available for all donors in the two sets of samples to allow means and SD values to be calculated. Population model parameters are detailed in Table S3.


(3) $$FC_{gene_{i}}={\left[gene_{i}\right]}_{disease}∕{\left[gene_{i}\right]}_{healthy}$$

- **Exposure to budesonide and cyclosporine.** Clinical PK data in PBC were available for two immunomodulators, budesonide [26] and cyclosporine A [27,28]. Cyclosporine A compound file was available in the Simcyp library, while budesonide compound file, developed by the University of Manchester and evaluated in a Crohn’s disease population [29], was accessed in the Simcyp Member Area (https://members.certara.co.uk). Each simulation was conducted as a single dose (6 mg budesonide or 5 mg/kg cyclosporine) for 24 h in 10 trials with 10 virtual individuals in each trial (90 % female, age range: 20–60 years) at healthy baseline and in cholestatic liver disease.
- **Changes in bilirubin levels.** To simulate the impact of dysregulation of hepatobiliary transporters in cholestatic liver disease on serum bilirubin, bilirubin levels were simulated using a bilirubin model previously developed by AstraZeneca [30]. The compound files were accessed in the Simcyp Member Area. Several factors were considered in the model, as briefly described below. Full PBPK was used to enable inclusion of unconjugated bilirubin as a substrate of hepatic OATP1B1/3 and conjugated bilirubin as a substrate of hepatic OATP1B1/3, MRP2, and MRP3, while metabolism (conjugation) of bilirubin is mediated by hepatic UGT1A1. The model reflected activity of the UGT1A1*28 variant, and therefore, activity parameters were changed to reflect a baseline UGT1A1*1 activity, Vmax of 391 pmol/mg/min and Km of 3.1 μM [31], and these parameters were kept the same in both healthy and cholestatic liver disease models (reports indicate that bilirubin conjugation is largely preserved in PBC, while transport pathways are dysregulated [32]). Hepatic UGT1A1 expression variability was set to 30 %. Simulations were performed as a bilirubin infusion for one week to ensure equilibrium was reached for both conjugated and unconjugated bilirubin. Production of bilirubin, as an endogenous compound, was simulated as an intravenous infusion at a rate of 3.8 mg/kg/day [33], equivalent to 26.6 mg/kg over the simulation period. Each simulation was conducted in 10 trials with 10 virtual individuals in each trial (90 % female, age range: 20–60 years). Model output focused on changes in levels of steady-state total bilirubin. Simulated values were compared with those reported in clinical literature at healthy baseline based on large cohort studies [34,35] or in cholestatic liver disease using reported [36] and unpublished in-house data.

## 3. Results

### 3.1. Quality control and characterization of plasma-derived EVs

Visualization of EVs (Fig. 1b) and corresponding size distribution profiles (Fig. 1c) reflected an overall size range of 35–350 nm, with a number-weighted mean size of 152 nm in plasma from healthy donors and 162 nm in plasma from donors with cholestatic liver disease. Average concentration of EVs was 28.4 × 10^12^ particles/mL in healthy plasma (range: 12.5–40.5 × 10^12^ particles/mL plasma), compared with an average of 21.9 × 10^12^ particles/mL in plasma from donors with cholestatic liver disease (range: 16.7–31.7 × 10^12^ particles/mL plasma). Total protein content of EV samples (Fig. 1d) was 17.4 ± 0.9 mg/mL of plasma from healthy donors, compared with 14.5 ± 6.4 mg/mL of plasma from donors with cholestatic liver disease. RNA quality and yield were assessed (Fig. 1e), reflecting DV200 score of 53 % ± 4 % in healthy samples (55 % ± 8 % in cholestatic liver disease) and cfRNA yield of 7.8 ± 4.9 ng/mL of healthy plasma (5.0 ± 3.7 ng/mL in cholestatic liver disease). No statistically significant differences were observed between the two sets of samples in relation to EV size distribution, EV particle concentration, protein yield, and cfRNA yield and quality (Fig. 1b-e). Additional QC details are included in Table S1. EV proteomics (*n* = 562 quantified proteins in healthy EV samples) identified annexins A1, A2, A5 and A6 as evidence of mixed-size (small and large) plasma EVs (Fig S4a). Tetraspannin markers of small EVs (CD9, CD63 and CD81) were below the limit of quantification, while other markers of small EVs, such as CD44, CD59, HSP90 and LAMP1 were identified. Serum protein contaminants (apolipoproteins and serum albumin) were present in isolated EV samples, as expected with polymer-based enrichment methodology (Fig S4b). There was no significant difference in EV shedding between the two sets of plasma samples (Fig S5), and therefore, shedding correction was not necessary for relative quantification.

### 3.2. Transcriptomic profile of plasma-derived EVs

**Fig. 2.**
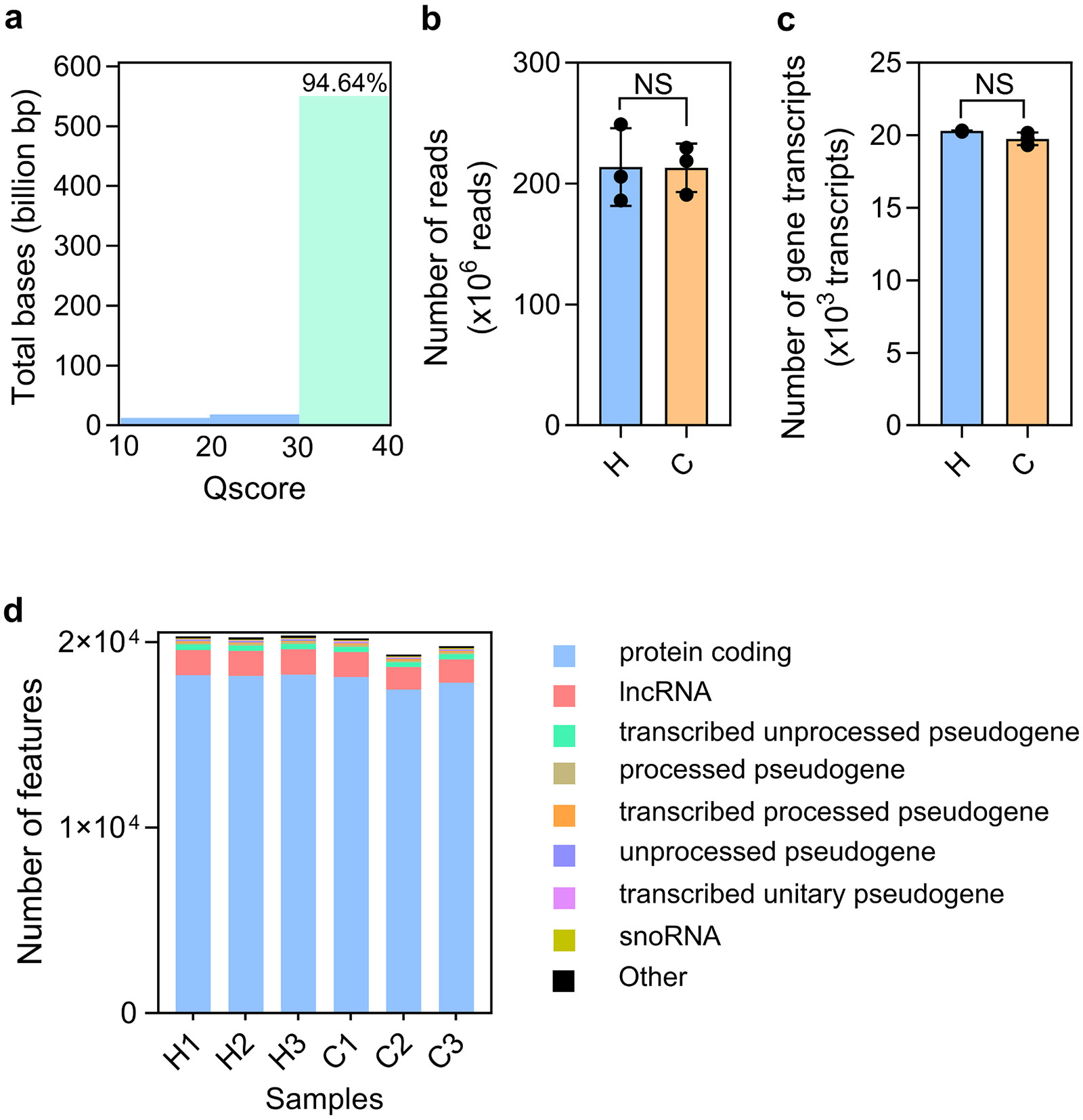
Sequencing data analysis and quality control. The quality of sequencing data (a) with the majority of bases (94.64 %) having Qscores of at least Q30, representing base call accuracy of at least 99.9 %. The total number (in millions) of reads (b) and the number of total mapped gene transcripts (c) are even across the samples. The number of transcript features identified in each sample (d) reflect a majority of protein-coding genes monitored across all samples (89.84 % ± 0.19 %), followed by long non-coding RNA (6.56 % ± 0.12 %). Other types of RNA identified include antisense RNA and microRNA. In panels b and c, data are plotted as mean and SD. Abbreviations: C, cholestatic liver disease; H, healthy; lncRNA, long non-coding RNA; snoRNA, small nucleolar RNA; NS, non-significant differences based on an unpaired *t*-test.

AmpliSeq achieved highly multiplexed targeted sequencing of approximately 20,800 human RefSeq genes, with over 18,300 protein-coding genes. Sequencing quality was excellent, with 94.64 % of sequenced bases achieving high quality scores (Qscores) of ≥ 30; i.e., ≥ 99.9 % base call accuracy (Fig. 2a). Between 82 % and 91 % of the sequenced reads were mapped to the reference genome, resulting in between 186.2 and 249.0 million mapped reads per sample (Fig. 2b), of which on average 73 % were uniquely mapped reads. The total number of transcripts monitored in all samples was 20,725. A quantitative output was generated for 19,320–20,180 transcripts in samples from donors with cholestatic liver disease, compared with a range of 20,240–20,329 in samples from healthy donors (Fig. 2c). The majority of transcripts were of protein-coding genes (89.8 % ± 0.2 % across all samples), followed by long non-coding RNA (6.6 % ± 0.1 %). Other types of monitored RNA included antisense RNA and microRNA, each at < 0.1 % of transcripts (Fig. 2d). Monitored druggable targets represented approximately 6.3 % of annotated transcripts (*n* = 1302 drug targets).

### 3.3. Reproducibility of quantitative liquid biopsy output

**Fig. 3.**
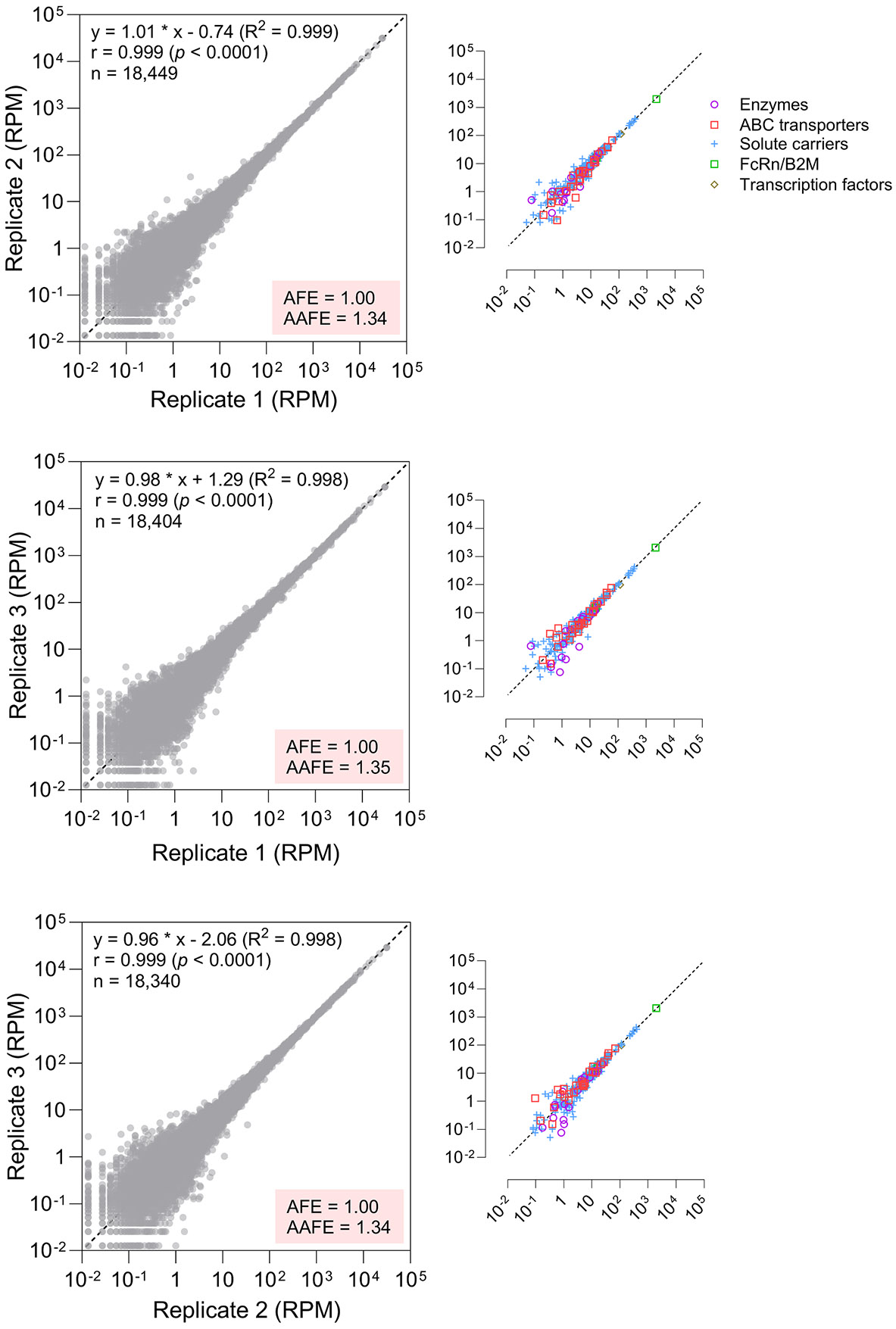
Reproducibility of liquid biopsy-RNAseq assay in a pool of healthy plasma cfRNA (*n* = 3) prepared and analyzed in triplicate. Agreement among EV transcriptome expression measured in the replicates was assessed using Pearson’s correlation (r), linear regression (R^2^) and fold error in measurements (AFE, AAFE). AFE is a measure of bias and AAFE is a measure of scatter of data. The expression of a subset of genes encoding for enzymes, ABC transporters, solute carriers (SLCs), FcRn and transcription factors relevant to the disposition and exposure to drugs and xenobiotics was compared among the replicates. The dashed lines in the plots are the lines of identity (1:1 ratio). Abbreviations: ABC transporters, ATP-binding cassette transporters; AFE, average fold error; AAFE, absolute average fold error; FcRn, neonatal Fc receptor; RPM, reads per million.

The reproducibility of the quantitative output of the liquid biopsy-RNAseq method was assessed using a pool of three healthy samples prepared and analyzed in triplicate (Fig. 3). Correlation between replicate data was excellent (Pearson correlation coefficient, *r* = 1.00, *p* < 0.0001), and linear regression reflected 1:1 quantitative ratio (R^2^ = 1.00). Bias and scatter of the data were assessed using average fold error (AFE) and absolute average fold error (AAFE), and the replicates reflected no bias (AFE = 1.00) and limited scatter (AAFE = 1.34). These results confirm the robustness of the method and its suitability for assessing changes in gene expression due to disease and other conditions or following a therapeutic intervention.

### 3.4. Global changes in expression in cholestatic liver disease

**Fig. 4.**
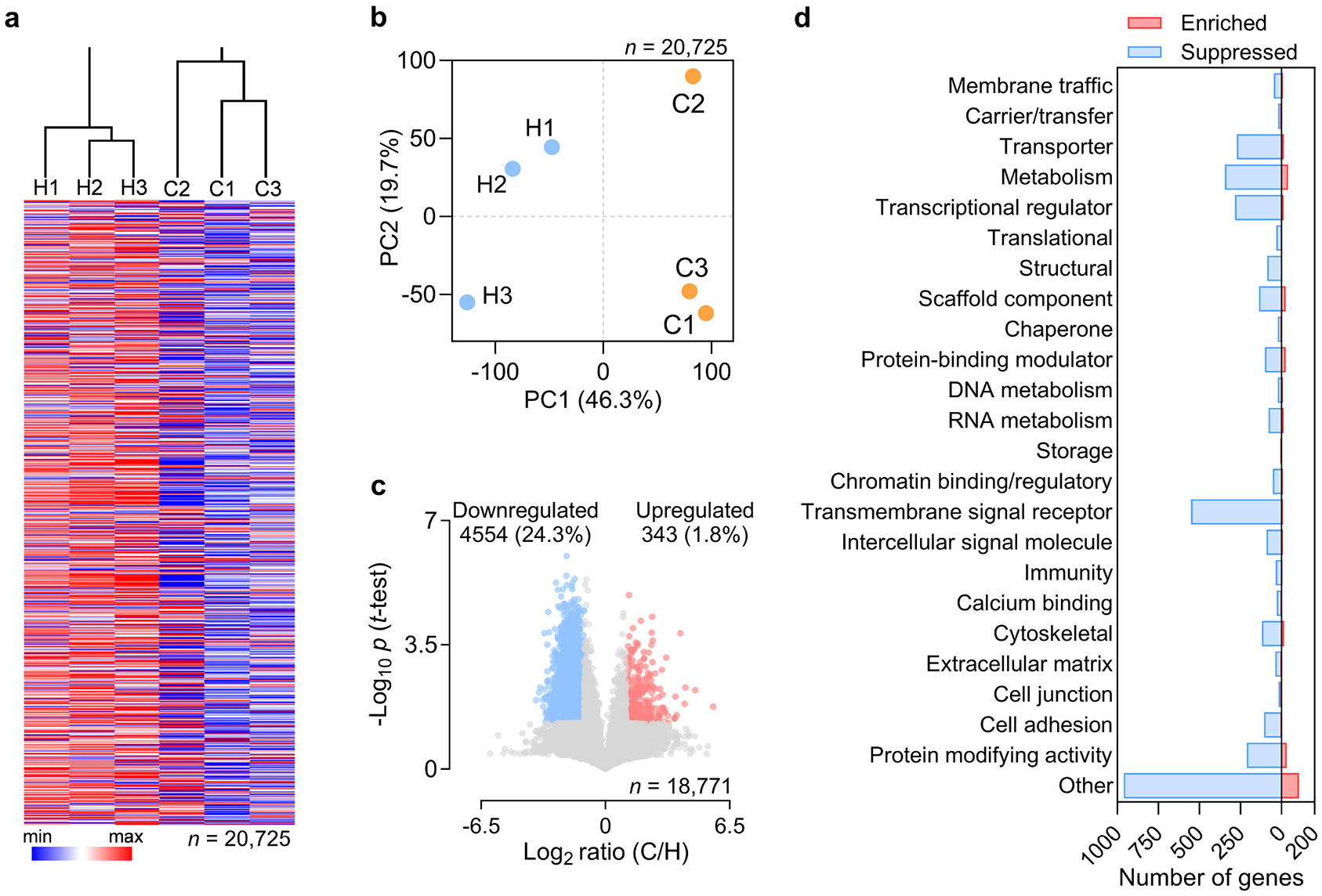
Differential expression profiles of EV samples from cholestatic liver disease relative to healthy controls. Heatmap of natural log-transformed expression data (*n* = 20,725 genes) clustered into two distinct groups following HCA based on Euclidean distance between values (a). PCA of expression data distinguished between the two groups, indicating that the variance component explained by the disease was 46.3 % (b). Volcano plot representing differential gene expression relative to healthy control identified up- and down-regulated genes (significance criteria: > 3-fold change, *p* < 0.05 based on an unpaired *t*-test) (c). Gene Ontology classification of enriched and suppressed genes in cholestatic liver disease based on differential expression analysis (d). Abbreviations: C, cholestatic liver disease; H, healthy; HCA, hierarchical cluster analysis; PCA, principal components analysis. PC, principal component.

Comparison of the expression of > 20,000 genes between the two sample sets by multivariate analysis is shown in Fig. 4. HCA applied to a heatmap of the data showed two distinct sample clusters reflecting healthy condition and cholestatic liver disease, with the healthy set showing more uniform expression profiles (Fig. 4a). PCA applied to the same dataset reinforced this observation and revealed that approximately 46 % of the variance among the samples (reflected by PC1) can be explained by the disease (Fig. 4b). Expression trends in cholestatic liver disease relative to healthy control suggested that there was an overall downregulation effect on gene expression (Fig. 4c). Most of the affected genes, based on Gene Ontology annotation, belonged to metabolic (enzymes), transport (transporters and carriers), transmembrane signaling and transcription regulation pathways (Fig. 4d).

### 3.5. Expression of enzymes, transporters and transcription factors

**Fig. 5.**
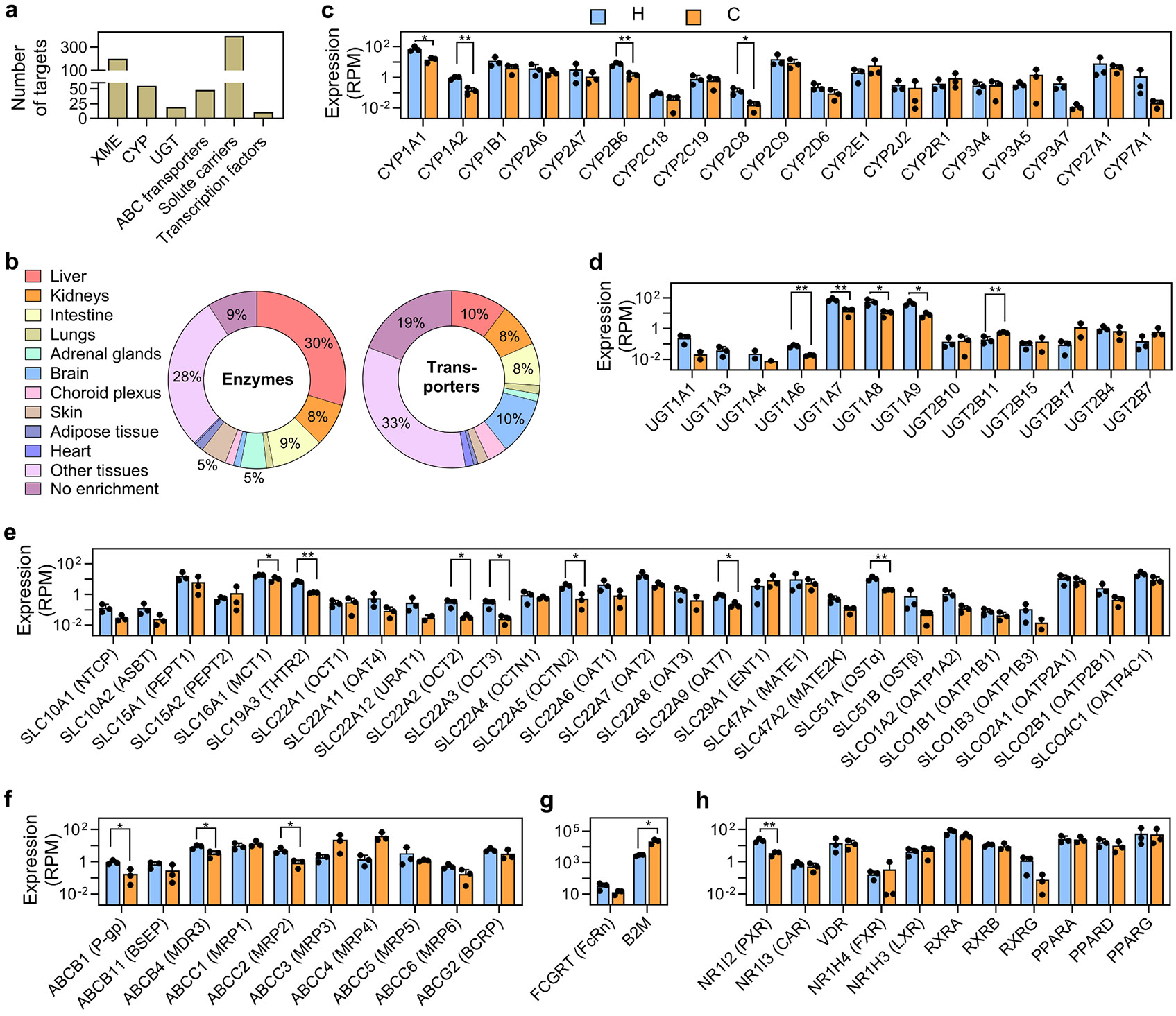
Differential expression of enzymes, transporters and transcription factors relevant in drug development. A breakdown of the numbers of xenobiotic- and drug-metabolizing enzymes, transporters and transcription factors monitored in plasma-derived EVs (a). Tissue enrichment of enzymes and transporters, showing liver-enriched enzyme expression and ubiquitous expression of transporters (b). Differential expression of CYP enzymes (c), UGT enzymes (d), solute carriers (e), ABC transporters (f), FcRn subunits (g), and transcription factors (h) in cholestatic liver disease relative to healthy controls. In panels c to h, data are plotted as mean and SD. Comparison of expression was carried out using an unpaired *t*-test; *, *p* < 0.05; **, *p* < 0.01. Abbreviations: C, cholestatic liver disease; CYP, cytochrome P450; FcRn, neonatal Fc receptor; H, healthy; UGT, glucuronosyltransferase; XME, xenobiotic-metabolizing enzyme; ABC transporter, ATP-binding cassette transporter; RPM, reads per million.

A total of 199, 444 and 11 gene transcripts of XMEs, transporters, and transcription factors, respectively, were quantifiable in plasma-derived EVs; a detailed list is included in Table S2. The genes included 55 CYP enzymes, 19 UGT enzymes, 48 ABC transporters and 396 solute carriers (Fig. 5a), in addition to FcRn (relevant to the disposition of mAbs). Moreover, 78 of these pathways are also molecular drug targets (Table S2). Approximately half (47 %) of the monitored enzymes were enriched in organs relevant to drug elimination (particularly, the liver, kidneys and intestine), whereas monitored transporters reflected more ubiquitous expression, with approximately 26 % enriched pathways (Fig. 5b). Assessment of disease-driven changes focused on expression of CYP enzymes (Fig. 5c), UGT enzymes (Fig. 5d), solute carriers (Fig. 5e), ABC transporters (Fig. 5f), FcRn subunits (Fig. 5g), and transcription factors (Fig. 5h), relevant in drug development, which were monitored across the two conditions. Differentially expressed enzymes (*t*-test, *p* < 0.05) included CYPs 1A1, 1A2, 2B6, 2C8; CES2; FMO5; GSTs A1, A3, M2; MGST1; SULTs 1A1, 1A2, 1B1, 1C3, 2B1, 4A1; and UGTs 1A6, 1A7, 1A8, 1A9, 2B11. Differentially expressed drug transporters (*t*-test, *p* < 0.05) included ABCB1 (P-gp/MDR1), ABCB4 (MDR3), ABCC2 (MRP2), SLC16A1 (MCT1), SLC19A3 (THTR2), SLC22A2 (OCT2), SLC22A3 (OCT3), SLC22A5 (OCTN2), SLC22A9 (OAT7), and SLC51A (OSTα). The listed PK-relevant targets were downregulated, except FMO5 and UGT2B11. In addition, the transcription factor, NR1I2 (PXR), was differentially expressed in cholestatic liver disease (fold change of 0.2, *t*-test, *p* < 0.01).

### 3.6. Simulated budesonide and cyclosporine levels in cholestatic disease

**Fig. 6.**
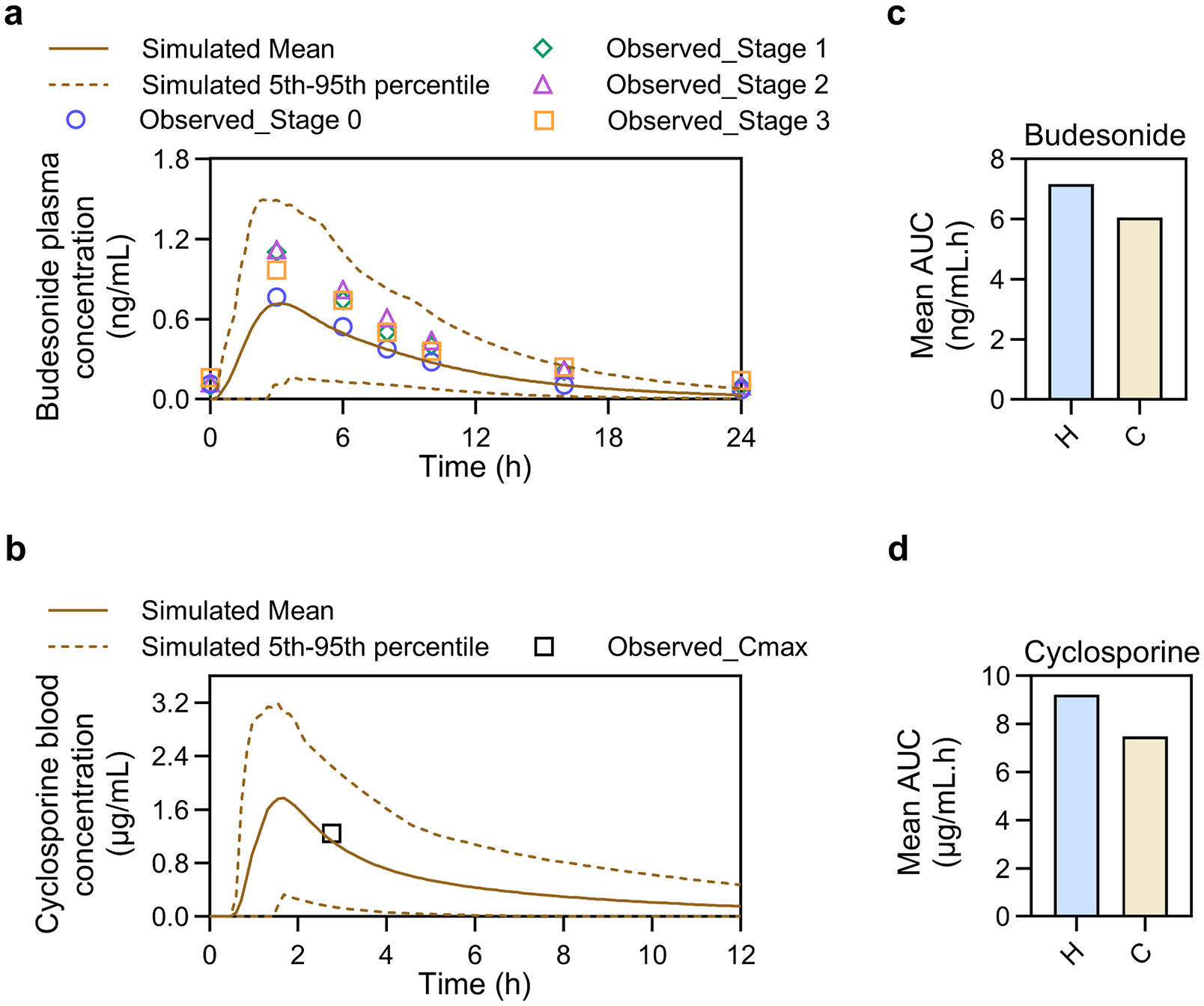
Simulated plasma budesonide and blood cyclosporine A concentrations compared with observed mean values in cholestatic liver disease (a). The mean AUC values from simulations using the two models were compared, predicting no significant change in exposure to CYP3A substrates in cholestatic liver disease (b). Observed data for PBC from Rautiainen et al., 2006 [26] (budesonide) and Robson et al., 1984 [27] and Beukers et al., 1992 [28] (cyclosporine). Abbreviations: C, cholestatic liver disease; Cmax, maximum concentration; H, healthy; AUC, area under the drug concentration-time curve; PBC, primary biliary cholangitis.

Simulated drug concentration-time profiles were in overall agreement with mean clinical drug concentrations for both budesonide and cyclosporine, and the levels were consistent with early-stage cholestatic liver disease (Fig. 6a,b). Mean predicted area under the curve (AUC) was modestly different from healthy exposure, reflecting a mean reduction of 16 % and 19 % for budesonide and cyclosporine, respectively (Fig. 6c, d), possibly attributed to lack of change in CYP3A kinetics and slightly reduced binding of the two substrates to serum albumin. The data suggest that patients with PBC may receive a typical dose for patients with normal liver function in the case of CYP3A substrates.

### 3.7. Simulated changes in serum bilirubin in cholestatic liver disease

**Fig. 7.**
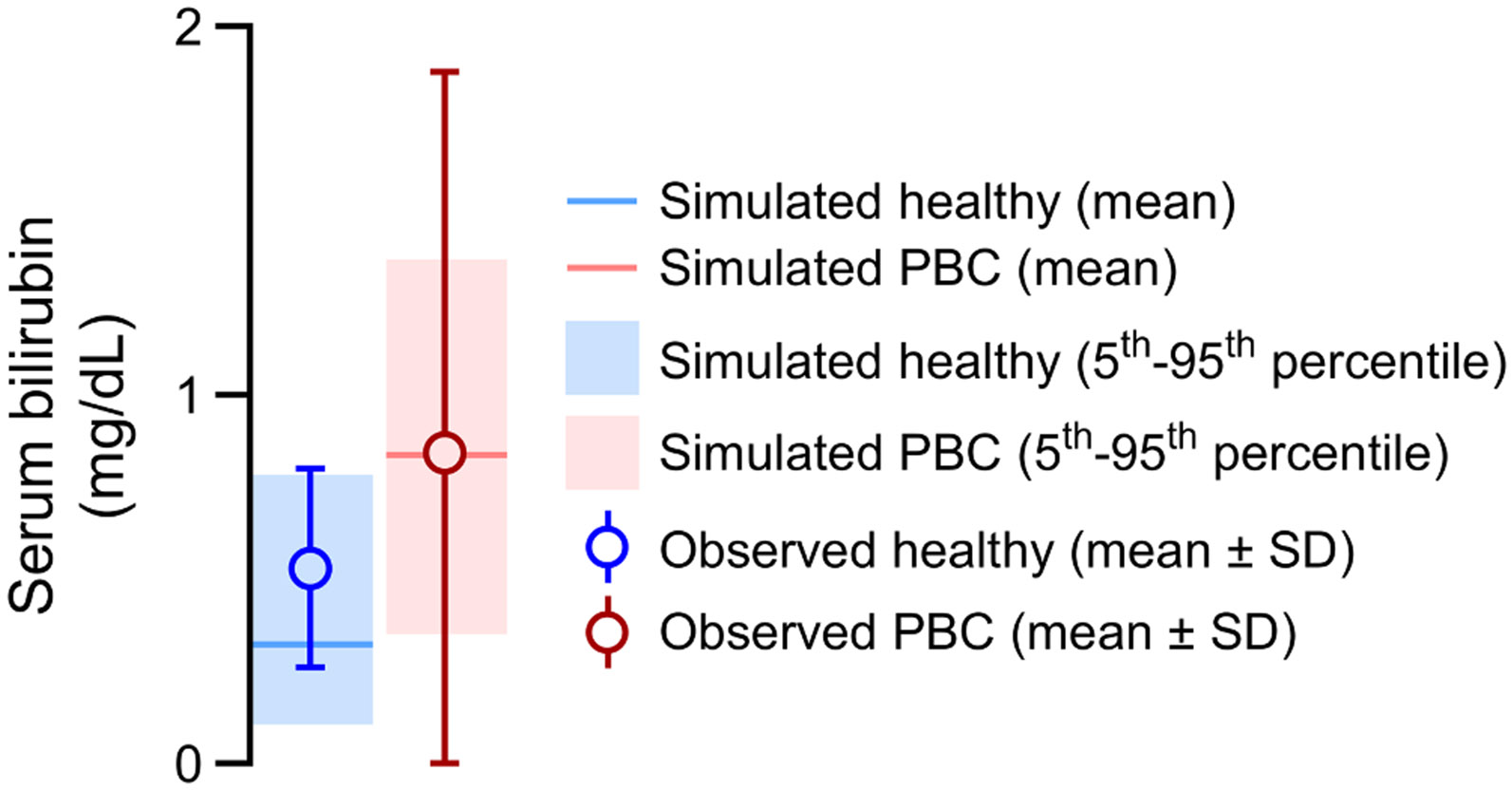
Simulated and observed total serum bilirubin in cholestatic liver disease relative to healthy controls. Observed data from Zucker et al. (2004) [34] and Sudlow et al. (2017) [35] for healthy baseline and from Ghonem et al. (2020) [36] and other in-house data for PBC. Abbreviations: PBC, primary biliary cholangitis.

Simulated levels of total serum bilirubin were 0.32 ± 0.23 mg/dL (range: 0.05–1.71) and 0.84 ± 0.36 mg/dL (range: 0.22–2.27) in healthy and cholestatic liver disease models, respectively. Observed values were within the simulated ranges, measuring 0.54 ± 0.27 and 0.84 ± 1.04 mg/dL, respectively (Fig. 7). While the model output indicated an increase in mean total bilirubin levels in cholestatic liver disease, overlap was extensive between bilirubin ranges. Simulated direct bilirubin accounted for 18 % ± 9 % and 62 % ± 13 % of total bilirubin in healthy and cholestatic liver disease simulations, respectively, indicating increased ratio of direct-to-total bilirubin relative to healthy baseline, in line with previous reports of the ratio rising to 30 % in early stages of cholestatic liver disease, and to 70 % in later stages and in liver cirrhosis [37].

## 4. Discussion

A recent US Food and Drug Administration [38] guidance-for-industry document warned that different subgroups of patients are often excluded by default from clinical testing of new drugs without a clinical or scientific rationale. A consequence of such exclusion is that changes in dosing recommendations for patient subgroups become available only after regulatory approval. While the FDA’s call for broadening enrollment eligibility criteria aims to increase the diversity of clinical trial populations, practical implementation remains challenging, particularly the prerequisite of adequate patient characterization prior to dosing. Characterization of pathways that define exposure of an individual patient to a certain drug should go beyond genotyping to achieve a quantitative prediction of the liver’s metabolic and elimination capacity. While liver biopsy is invasive, and therefore not practical for routine clinical practice, the use of tissue proteomics to inform PBPK modelling was previously demonstrated to achieve precise dosing in a cohort of obese patients [39], highlighting the utility of ‘omics’ in evaluating drug exposure. We recently developed an individual dosing approach guided by minimally invasive liquid biopsy measurements in a cohort of patients with renal impairment and demonstrated a 3-fold reduction in drug exposure variability [13]. The developed liquid biopsy assay was also shown to correlate with the *in vivo* activity of cytochrome P450s and P-gp in a hospitalized cohort with cardiovascular disease [9]. Hospitalized patients and those with chronic disease tend to suffer from varying grades of comorbidities and are especially susceptible to drug interactions. These complex disease-drug and drug-drug interactions necessitate the use of PK models compatible with quantitative characterization methods to better predict drug response [2]. The present report aims to provide a technical account for the deployment of a liquid biopsy-informed PBPK modelling approach to assess changes in clinical drug exposure. A proof-of-concept is demonstrated with cholestatic liver disease based on a small set of samples (*n* = 3). The work builds and expands upon methodological research in the area of EVs, published in this journal [40,41] and elsewhere [10,12].

The use of liquid biopsy in translational pharmacology, beyond cell-free DNA (cfDNA) characterization [8], relies on the extraction and quantification of EV-derived cfRNA or protein, and linking such measurements with tissue health and functional capacity by monitoring expression of pathways enriched in organs relevant to drug metabolism and disposition, particularly the liver, kidneys and intestine [11]. Global proteomics of EVs did not yield useful data for the pathways of interest, possibly due to low abundance of such proteins in organ-shed EVs and the dominant mass spectrometric response of serum protein contaminants, such as albumin and immunoglobulins. RNA in serum tends to be degraded by nucleases [12], whereas cfRNA in EVs is protected by the vesicles and is subsequently amplified by PCR over at least 23 cycles before sequencing, which enabled monitoring of > 20,000 genes, including > 600 drug disposition pathways. Notably, approximately 50 % of monitored enzymes and 30 % of monitored transporters were enriched in the liver, kidneys or intestine.

EV-based measurements require normalization for variation in tissue-to-blood shedding, as previously described for the liver [12], except in cases where variability in shedding is limited. The liver shedding factor, proposed in our previous research [12], was revised to exclude markers involved in blood clotting, since their expression may be suppressed in liver disease [21]. Measured shedding in cholestatic liver disease was not different from healthy baseline, as reflected by the comparable particle concentrations, protein contents and RNA yields determined in the two sets of samples. These quality controls serve to ensure that the extracted analyte is usable for downstream analysis and that conclusions are not biased. EV size distribution in both sets of samples was consistent with a mixed population of large and small EVs, as expected with polymer-assisted precipitation, which tends to generate a high EV yield while sacrificing enrichment. Detailed notes on the different analytical steps and quality controls are shared in a detailed protocol in the Supplement.

Measurements in liquid biopsy can be used to complement genotype/phenotype information as described previously [2], with the advantage of broad coverage of a multitude of pathways offered by liquid biopsy measurements. To illustrate this point within the context of drug metabolism and disposition, genome-wide screening of EVs allowed monitoring of 199 XMEs, 444 transporters and FcRn subunits (relevant to disposition of mAbs [42]), in addition to 11 transcription factors, consistent with the wide coverage reported in our previous applications [9,12,13]. Estimation of disease perturbation in expression from healthy baseline was possible for 93 % of monitored PK pathways. Disease-to-healthy ratios are used in PBPK models to estimate changes in intrinsic clearance of drugs metabolized and/or transported by the perturbed pathways. In addition to PK pathways, the screening also allowed monitoring of 1302 molecular drug targets, relevant to predicting drug response changes.

Individual-level expression and disease perturbation data, such as those presented herein, are essential systems parameters for building sub-population and virtual twin models within a model-informed precision dosing framework [43]. Indeed, the preliminary cholestatic liver disease model we developed in this study to reflect PBC (based on expression changes in enzymes and transporters, perturbation in serum albumin levels, and differences in demography) reflected clinical levels of the immunomodulators, budesonide [26] and cyclosporine [27,28], and predictions were consistent with early stages of the disease. Once established in larger sample sets, these trends may be used to guide clinical dosing recommendations in patient sub-populations. The bilirubin model was based on a healthy model previously published by AstraZeneca [30], which was modified to reflect changes in cholestatic liver disease. The model reflected an increase in total bilirubin mainly driven by increased levels of conjugated bilirubin, in line with previous clinical observations [37]. Disease-driven changes in expression of OATP1B1 (reduced), MRP2 (reduced) and MRP3 (increased), highlight that reduced biliary elimination by MRP2 may be compensated by increased transport into the blood by MRP3. Tissue-level evidence for lower OATP1B [44], lower MRP2 [45,46] and higher MRP3 [47] expression in biliary disease corroborates such trends. This reinforces that transporter dysregulation in cholestatic liver disease may be a key factor contributing to increased levels of conjugated bilirubin in the blood.

It should be emphasized that, due to the small sample size, the work presented in this report is a proof-of-concept exploratory analysis to demonstrate feasibility. The key message from the examples explored herein may be highlighted in the potential use of liquid biopsy-derived systems data and physiologically relevant PK models in predicting patient exposure and guiding dosage changes in disease sub-populations. However, the research presented herein is intended only to describe the methodology for generating and applying the data and quality controls required to ensure the usability of such data, while any clinical conclusions are only preliminary and will require further confirmation with a larger sample set.

## Supplementary Material

1

## Figures and Tables

**Table 1:** Demographics of plasma donors. Plasma samples were collected from healthy donors (*n* = 3) and donors with cholestatic liver disease (*n* = 3).

	Healthy	Cholestatic liver disease
**Sex**	F (3)	M (1), F (2)
**Age (years)**	52.3 ± 2.5	68.0 ± 5.3
**Ethnicity**	Hispanic (1), Black (2)	White
**Smoking status**	No	No
**BMI (kg/m^2^)**	29.7 ± 7.8	26.0 ± 4.9

Age at blood collection; F, female; M, male. Age and BMI are expressed as mean ± SD.

## Data Availability

The raw data generated in this study are deposited on the NCBI GEO database under accession number GSE302189. The read count data are available on request from the corresponding author.

## Appendix A. Supporting information

Supplementary data associated with this article can be found in the online version at doi:10.1016/j.jpba.2025.117244.
