# Supplementary material for: A liquid biopsy-RNAseq method for monitoring the expression of genes involved in drug disposition: Proof-of-concept application to cholestatic liver disease
Source: J Pharm Biomed Anal. Author manuscript; Available in PMC 2026 Jul 6. (PMC13335542; doi:10.1016/j.jpba.2025.117244)
Supplement: 1 [file NIHMS2186303-supplement-1.docx]

**Supplementary Information**

**A Liquid Biopsy-RNAseq Method for Monitoring the Expression of Genes Involved in Drug Disposition: Proof-of-Concept Application to Cholestatic Liver Disease**

Amit Dahal, Teresa Sierra, Colleen M. Hayes, David N. Assis, Amin Rostami-Hodjegan, Nisanne S. Ghonem, Brahim Achour*

*Correspondence to: Dr. Brahim Achour (email: [achour@uri.edu](mailto:achour@uri.edu))

**Supplemental Content**

**Protocol, Notes and QC Steps**

**Figure S1.** Extracted EV pellet and expected biomolecular cargo.

**Figure S2.** Assessment of the quality and quantity of cfRNA isolated from plasma-derived EVs.

**Figure S3.** Assessment of the size and quality of cDNA libraries.

**Figure S4.** Identified markers and contaminants of isolated EVs from healthy plasma samples.

**Figure S5.** Shedding factor measured in plasma samples from healthy donors and donors with cholestatic liver disease.

**Table S1.** Experimental parameters used as quality controls for the sequencing of cfRNA from plasma-derived EVs.

**Table S2.** Expression of enzymes, transporters and transcription factors in plasma EVs from healthy donors and donors with cholestatic liver disease.

**Table S3.** Cholestatic liver disease population model parameters.

**Protocol, Notes and QC Steps**

**Preparation of solutions and buffers**

- Prepare fresh 80% molecular biology grade ethanol with nuclease-free water
- Prepare fresh 70% molecular biology grade ethanol with nuclease-free water
- Prepare fresh 1XPBS buffer by dilution with nuclease-free water
- Prepare Wash Solution 1; add 34 mL 100% molecular biology grade isopropanol to the 66 mL of MagMAX™ Total Nucleic Acid Wash Solution Concentrate
- Bring the MagMAX™ Cell-Free Total Nucleic Acid Magnetic Beads to room temperature and vortex beads thoroughly
- Bring Agencourt AMPure XP beads to room temperature, vortex beads thoroughly

**Plasma preparation**

- Thaw plasma samples on ice (or overnight in ice), making sure samples are completely thawed before processing
- Vortex mix samples thoroughly, then centrifuge at 3,000 g for 15 min at 4°C. Transfer the supernatant into a fresh 15 mL conical tube

**Extraction of extracellular vesicles (EVs) from plasma**

- Add 1 mL plasma to a 15 mL conical tube
- Add 0.5 mL 1X PBS to plasma; vortex mix briefly
- Add 50 μL Proteinase K. NOTE: use proteinase step only when extracting nucleic acids. Do not use with proteomics
- Incubate at 37°C for 10 min
- Cool down at 4°C for 20 min
- Add 0.39 mL of ExoQuick reagent. NOTE: a 1:4 reagent:sample volume ratio must be maintained
- Mix well by inverting or flicking the tube. NOTE: do NOT vortex mix
- Incubate at 4°C for 1 h. The tubes should not be shaken or mixed during the incubation period and should remain upright
- Centrifuge at 1,500 *g* for 45 min at 4°C.
- **QC1:** after centrifugation, EVs may appear as a beige/white pellet at the bottom of the tube (**Fig S1**)
- Discard supernatant by pipetting. Spin down residual ExoQuick reagent by centrifugation at 1500 *g* for 5 min. Remove all traces of fluid by aspiration, taking great care not to disturb the precipitated EVs in the pellet. NOTE: you can wash the pellet gently with 1X PBS and discard the supernatant
- Resuspend the pellet in 200 µL using sterile 1X PBS, or specific buffer according to your downstream application. Use the precipitated EVs immediately. Do not freeze for future use
- Apply a DNAse step to remove DNA in EVs suspension
- **QC2:** visualization of EVs by transmission electron microscopy (TEM) (**Fig 1b**)
- **QC3:** determination of EVs size distribution nanoparticle tracking analysis (NTA) (**Fig 1c**)
- **QC4:** determination of protein content/yield of extracted EVs using a BCA assay (**Fig 1d**)

**Extraction of cell-free RNA (cfRNA) from EVs**

- Make up volume to 2 mL with 1X PBS
- Combine the 2 mL resuspended EV pellet with 2.5 mL of MagMAX Cell-Free Total Nucleic Acid Binding Buffer. NOTE: a 1:1.25 sample:binding buffer volume ratio must be maintained
- Add 60 μl of magnetic beads to the tube. NOTE: make sure the beads are mixed thoroughly and equilibrated to room temperature before use
- Shake tube at 1000 rpm for 10 min at room temperature to extract and bind cfRNA on the beads
- Place tube on a Dynamag-15 magnetic stand for 5 min. NOTE: do not rush the bead collection step, as loss of beads will reduce yield
- Carefully remove and discard supernatant, without disturbing the bead pellet
- Resuspend the beads in 1 mL of Wash Solution 1, then mix by pipetting up and down carefully, ensuring all beads are released from the tube walls
- Transfer the bead slurry to a new 1.5 mL microcentrifuge tube and place on the DynaMag™–2 Magnet stand for 20 seconds. Do not discard the 15-mL conical tube
- Collect the supernatant from the bead pellet in the 1.5-mL tube and use it to rinse remaining beads from the 15 mL conical tube
- Carefully transfer the supernatant back to the 1.5 mL tube on the DynaMag™–2 Magnet stand
- Leave the 1.5 mL tube on the magnet stand for 2 min or until solution clears, then discard the supernatant, being careful not to disturb the bead.
- Add 1 mL of 80% ethanol (prepared beforehand using nuclease-free water and molecular biology grade ethanol)
- Vortex thoroughly, then centrifuge briefly to collect the bead solution
- Place on DynaMag™–2 Magnet stand for 2 min, or until solution clears
- Discard the supernatant
- Repeat alcohol wash step for a second wash with 1 mL of 80% ethanol
- Carefully remove and discard the ethanol
- Air-dry the beads for 5 min, then remove any remaining ethanol with a 10 μL pipette
- Resuspend the beads in 400 μL of MagMAX™ Cell-Free Total Nucleic Acid Elution Solution and vortex for 5 min at high speed
- Centrifuge briefly to collect the solution, then place the tube on the DynaMag™–2 Magnet stand for 2 min to capture beads
- Transfer the supernatant to a new 1.5 mL microcentrifuge tube

**Concentrating extracted cfRNA**

- Add 500 μL of MagMAX™ Cell-Free Total Nucleic Acid Lysis/Binding Solution to the 1.5 mL microfuge tube. NOTE: a 1:1.25 sample:binding buffer volume ratio must be maintained
- Add 10 μL of well-vortexed MagMAX™ Cell-Free Total Nucleic Acid Magnetic Beads. NOTE: make sure the beads are mixed thoroughly before use. Do not use less than 10 μL of beads suspension for the rebinding step or loss of analyte may occur
- Vortex the tube at high speed for 5 min to bind nucleic acids to the beads
- Briefly centrifuge the tube to collect the volume, and then place the tube on DynaMag™–2 Magnet stand for 5 min to capture the beads
- Discard the supernatant
- Add 1 mL of Wash Solution 1
- Vortex briefly, then centrifuge briefly to collect the volume
- Place the tube on DynaMag™–2 Magnet stand for 2 min
- Discard the supernatant
- Add 1 mL of 80% ethanol
- Vortex the tube briefly, then centrifuge the tube briefly
- Place on DynaMag™–2 Magnet stand for 2 min, or until solution clears
- Discard the supernatant
- Repeat the alcohol wash step. Remove any remaining ethanol. NOTE: be careful to remove all the ethanol
- Air-dry the beads on the DynaMag™–2 Magnet stand for 3 min, taking care not to over-dry the beads
- Discard any remaining liquid
- Add 15 μL of MagMAX™ Cell-Free Total Nucleic Acid Elution Solution and shake on high speed for 5 min
- Briefly centrifuge, then place the tube on DynaMag™–2 Magnet stand for 2 min to capture the beads
- Transfer the eluate to a fresh 1.5 mL microfuge tube
- Store the purified cfRNA on ice for immediate use. Alternatively, the purified cfRNA can be placed at −20°C or −80°C for long-term storage. NOTE: aliquot to avoid excessive freeze-thawing of extracted cfRNA. Once thawed, do not reuse the RNA aliquots
- **QC5:** determination of the yield and quality of extracted RNA using an Agilent 2100 Bioanalyzer (**Fig S2**). Determine cfRNA concentration (this should be >100 pg/µL) and DV200 score (% of RNA fragments longer than 200 nt; this should be >30%) (**Fig 1e**). Also be alert to any genomic DNA peaks, which tend to appear as a large, high molecular weight peak or smear. DNA peaks were absent form out cfRNA samples (**Fig S2**). DNA contamination can interfere with accurate quantification of gene expression levels, especially for low-abundance transcripts

**Reverse transcription of cfRNA**

- Thaw cfRNA on ice immediately before use. Invert to mix and centrifuge briefly to collect the volume. Keep on ice during the procedure
- Using the Ampliseq cDNA Synthesis for Illumina Kit, combine in one well of 96-well PCR plate: 1 µL of 5X AmpliSeq cDNA Reaction Mix, 0.5 µL of 10X AmpliSeq RT Enzyme Mix and 3.5 µL of cfRNA from each sample. This should result in 5 µL total volume
- Seal the plate, vortex thoroughly, and centrifuge briefly
- Place on a thermal cycler, and run the RT program: choose the preheated lid option and set to 105°C, set the reaction volume to 5 µL, 42°C for 30 min, 85°C for 5 min, then hold at 10°C

**PCR amplification of cDNA**

- Use the AmpliSeq Transcriptome Human Gene Expression Panel and AmpliSeq HiFi Mix (Illumina) for this step
- To each well containing 5 µL of cDNA from the reverse transcription step, add 4 µL of 5X AmpliSeq HiFi Mix, 8 µL of 2.5X AmpliSeq Transcriptome Human Gene Expression Panel, 3 µL of nuclease-free water. NOTE: HiFi Mix is viscous so pipette slowly
- Pipette to mix, seal the plate, and centrifuge briefly to collect the volume
- Place on the thermal cycler and run the amplification program: choose the preheated lid option and set to 105°C, set the reaction volume to 20 µL, start at 99°C for 2 min, then 16 cycles of: 99°C for 15 seconds, 60°C for 16 min, and hold at 10°C
- NOTE: the Illumina amplification protocol is for 10 ng high-quality material, and it uses 12 cycles of amplification. Since the quality and quantity of RNA extracted from EVs are low, 4 cycles are added to the 12 cycles

**cDNA library preparation and sequencing**

- Libraries are prepared for sequencing using AmpliSeq Library PLUS (96 reactions)
- After cDNA amplification, briefly centrifuge to collect the volume and unseal the PCR plate
- To partially digest cDNA amplicons, add 2 µL of FuPa reagent to each well, seal the plate, vortex mix and centrifuge briefly
- Place the plate on the thermal cycler, and run the FUPA program: Choose the preheated lid option and set to 105°C, set the reaction volume to 22 µL, then start at 50°C for 10 min, 55°C for 10 min, 62°C for 20 min and hold at 10°C for up to 1 h
- Partial digestion is followed by ligation with Index 1 (i7) and Index 2 (i5) adapters, premixed in a single-use plate to ensure unique combinations. NOTE: Each library will have a unique index combination for dual-index sequencing
- Briefly centrifuge the plate to collect the content, and unseal
- Add the following in the order listed to each well: 4 µL Switch Solution, 2 µL AmpliSeq CD or UD Indexes for Illumina, and 2 µL DNA ligase. NOTE: To avoid library prep failure, do not combine these components but add them sequentially, making sure DNA ligase is added last. Switch Solution and DNA ligase are viscous so pipette slowly
- Seal the plate, vortex, and centrifuge briefly to collect the volume
- Place the plate on the thermal cycler, and run the LIGATE program: choose the preheated lid option and set to 105°C, set the reaction volume to 30 µL, start at 22°C for 30 min, 72°C for 5 min, and hold at 10°C for up to 24 h
- Library cleanup uses Agencourt AMPure XP beads (Beckman):
- Briefly centrifuge the plate, and unseal
- Add 30 µL AMPure XP beads to each well and seal the plate. NOTE: make sure the beads are mixed thoroughly and equilibrated to room temperature
- Vortex briefly and inspect wells to make sure the mixture is homogeneous. Centrifuge briefly and incubate at room temperature for 5 min
- Place on a magnetic stand for PCR plates until the mixture clears.
- While on the magnetic stand, unseal the plate and remove and discard the supernatant
- While on the magnetic stand, wash the beads twice with 150 µL of freshly prepared 70% molecular biology grade ethanol, incubate at room temperature until the solution is clear, discard the supernatant
- Seal the plate and centrifuge briefly
- Place on the magnetic stand, and unseal the plate. NOTE: make sure the plate is returned to the same orientation on the magnetic stand
- Remove all residual ethanol with a pipette
- Air-dry on the magnetic stand for 10 min. Inspect, and continue to air-dry until no visible ethanol remains. NOTE: residual ethanol causes library prep failure
- For library amplification (7 cycles), remove the plate from the magnetic stand and add 50 µL of amplification master mix to each well, and seal the plate (the master mix is prepared by combining and thoroughly mixing 45 µL of 1X Lib Amp Mix and 5 µL of 10X Library Amp Primers)
- Vortex and centrifuge briefly. NOTE: amplification takes place with the beads in the wells
- Place on the thermal cycler, and run a second amplification program: choose the preheated lid option and set to 105°C, set the reaction volume to 50 µL, start at 98°C for 2 min, then 7 cycles of: 98°C for 15 seconds, 64°C for 1 min, and hold at 10°C for up to 24 h
- Perform a second cleanup of amplified libraries using Agencourt AMPure XP beads:
- Briefly centrifuge the plate, and unseal. Add 25 µL AMPure XP beads to each well and seal the plate. NOTE: this step adds beads to the beads already in the wells
- Vortex and centrifuge briefly
- Incubate at room temperature for 5 min
- Place on a magnetic stand and wait until the liquid is clear
- Unseal the plate and transfer the supernatant to a new PCR plate
- Add 60 µL AMPure XP beads to each well containing the transferred supernatant, and seal the plate
- Vortex and centrifuge briefly
- Incubate at room temperature for 5 min
- Place on the magnetic stand and wait until the liquid is clear
- Unseal the plate. Remove and discard the supernatant from each well. NOTE: the amplicon libraries are captured by the beads on the walls of the wells
- While on the magnetic stand, wash the beads twice with 150 µL of freshly prepared 70% molecular biology grade ethanol, incubate at room temperature until the solution is clear, discard the supernatant without disturbing the beads
- Use a pipette to remove and discard residual ethanol from each well
- Air-dry on the magnetic stand for 5 min
- Remove from the magnetic stand and add 30 µL Low TE (Tris-HCl EDTA) buffer to each well, and seal the plate
- Vortex briefly to disperse the beads, and centrifuge briefly to collect the contents
- Place on the magnetic stand and wait until the liquid is clear
- Unseal the plate and transfer 27 µL of the supernatant to a new PCR plate. NOTE: the supernatant contains the amplicon libraries
- **QC6:** determination of the quality of prepared cDNA libraries using a Bioanalyzer, Fragment analyzer or TapeStation. Determine cDNA size (this should be in the range 260-300 bp) and the presence of fragments (this should be minimal). These techniques can also be used to determine concentration of the libraries for normalization and pooling. Alternatively, the Qubit fluorometer or a qPCR method can be used. In the current protocol, we used an Agilent TapStation 4200 to determine library quality and size (**Fig S3** and **Table S1**) and qPCR with a Roche LightCycler 96 to determine library concentrations for normalization (**Table S1**)
- Based on the quantification method in QC6, dilute libraries with low TE to a normalized starting concentration of 2 nM
- Pool the libraries by combining 10 µL from each diluted library
- To denature libraries, mix 11 µL of 2 nM sample library pool with 8.5 µL of 0.2 N NaOH, vortex mix, centrifuge briefly to collect the contents and incubate at room temperature for 5 min. Denature PhiX control library using the same steps
- To dilute the denatured libraries, add 127 µL pre-load buffer, 17 µL resuspension buffer, with a 5% denatured PhiX control spike-in to achieve a 130 pM final loading concentration of pooled libraries
- The final denatured and diluted pool is then sequenced using an Illumina NovaSeq sequencer
- NOTE: prepare 0.2 N NaOH solution fresh. Addition of NaOH serves to denture the double-stranded cDNA libraries into single strands for sequencing. PhiX is a high-quality spike-in library used as sequencing control in Illumina protocols. PhiX is added to sample libraries to improve the quality of sequencing. For low diversity libraries, the PhiX control spike-in should be at least 5%.
- **QC7:** sequencing quality is determined by the percentage of bases with Q-scores ≥ Q30 (**Table S1** and **Fig 2a**), the distribution and depth of sequencing, typically represented by the number of reads per library across the pool of libraries (**Table S1** and **Fig 2b**), coverage of the genome (**Fig 2c**), and the percentage of protein-coding amplicons (**Fig 2d**). For good quality sequencing, %≥Q30 should be at least 75%. The depth should be at least 30M reads per library, and evenly distributed across libraries, to capture both high and low abundance transcripts with limited bias across samples. For good coverage, at least 20k genes should be monitored successfully. The proportion of gene-coding transcripts should be at least 80%.

**Figure S1.** Extracted EV pellet and expected biomolecular cargo. The EVs are visualized with electron microscopy, and their size distribution is evaluated with nanoparticle tracking analysis. Protein and RNA content and quality are assessed before RNAseq and proteomics. Abbreviations: cfDNA; cell-free DNA; cfRNA, cell-free RNA; EVs, extracellular vesicles; QC, quality control; RNAseq, RNA sequencing.

**
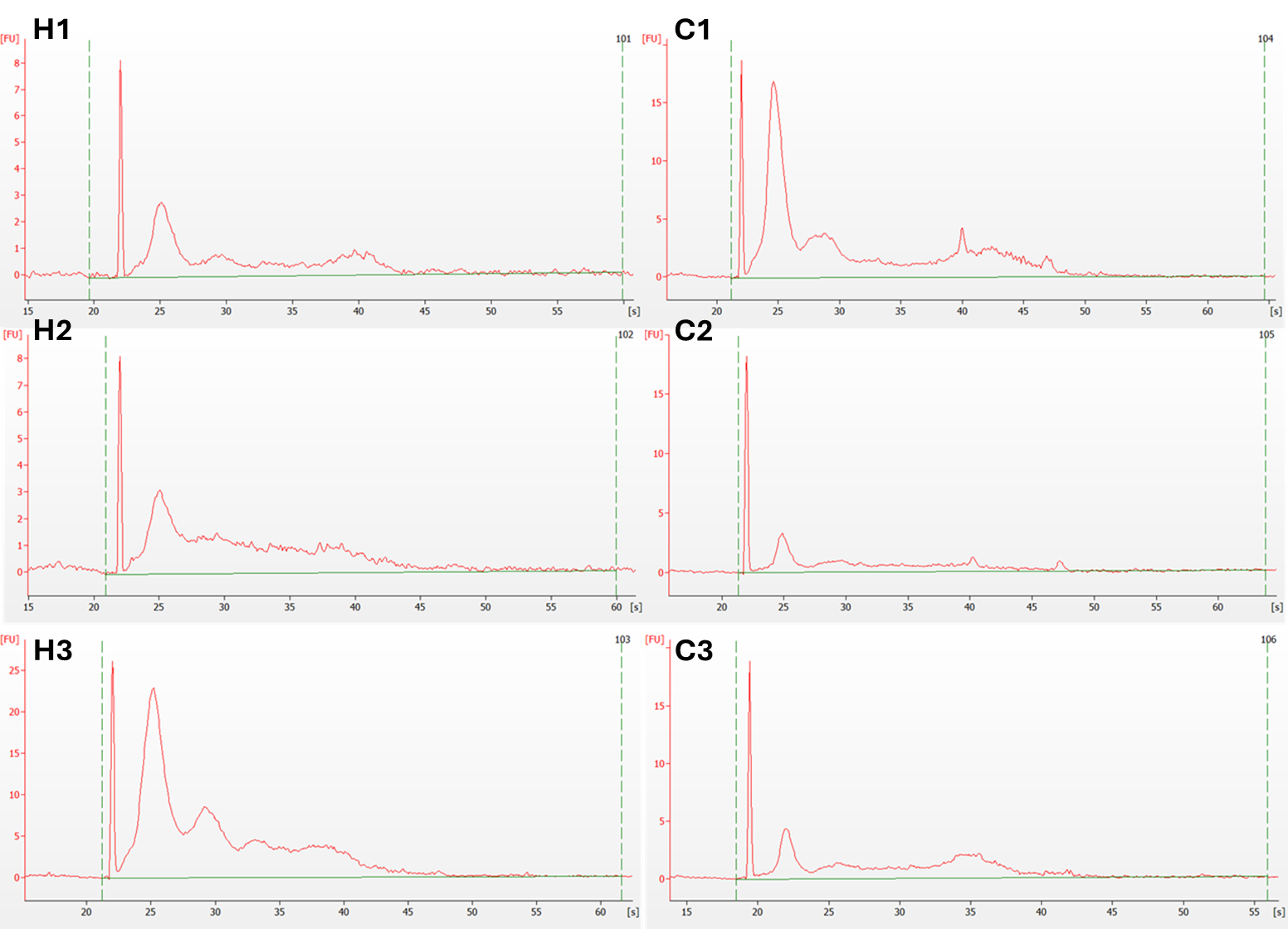
**

**Figure S2.** Assessment of the quality and quantity of cfRNA isolated from the plasma-derived EVs. The spectra show RNA peaks and the absence of genomic DNA.

**
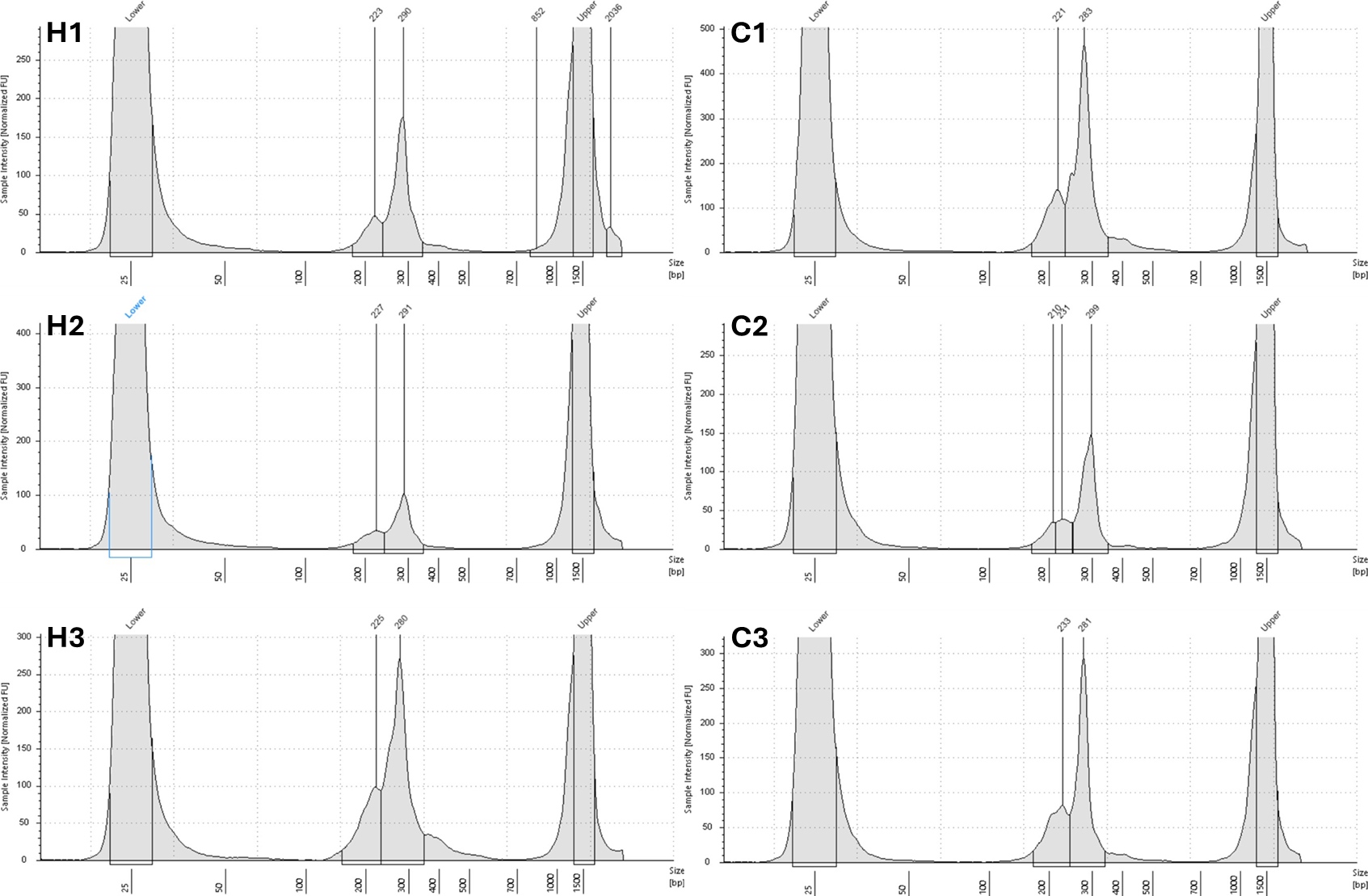
**

**Figure S3.** Assessment of the size and quality of cDNA libraries showing that all libraries had a size in the range 280-299 bp (mean size of 287 bp). The x-axis shows the size of the fragments, and the y-axis shows fluorescence intensity. Two markers (lower and upper) are shown on each spectrum with the analyte in the middle.

**Figure S4.** Identified markers (**a**) and contaminants (**b**) of isolated EVs from healthy plasma samples (n = 3). The markers are indicative of mixed-size EVs with enrichment of large vesicles. ANXA1/2/5/6 are markers of large EVs. CD44, CD59, HSP90 and LAMP1 are markers of small EVs. Typical markers of small EVs (CD9, CD63, CD81) were not detected. Data are presented as mean and SD.

**Figure S5.** Shedding factor measured in plasma samples from healthy donors and donors with cholestatic liver disease, reflecting lack of difference in shedding in the two sets of samples. Data are presented as mean and SD. Abbreviations: C, cholestatic liver disease; H, health; NS, non-significant differences based on an unpaired *t*-test.

**Table S1.** Experimental parameters used as quality controls (QCs) for the sequencing of cfRNA from plasma-derived EVs. QCs included cfRNA concentration and quality (DV200), cDNA size and concentration, sequencing depth and quality.

|  | **cfRNA** | | | **cDNA** | | **Sequencing** | | | | |
| --- | --- | --- | --- | --- | --- | --- | --- | --- | --- | --- |
| **Sample** | **[cfRNA] (ng/µL)** | **DV200 (%)** | **RNA library input (ng)** | **Library qPCR (nM)** | **Library size (bp)** | **Index** | **Lane 1 reads** | **Lane 2 reads** | **Total reads** | **%≥Q30** |
| **H1** | 0.238 | 50 | 0.8330 | 6.16 | 290 | GTGAATAT-CGCTATGT | 1.2E+08 | 1.3E+08 | 2.49E+08 | 94.64 |
| **H2** | 0.374 | 56 | 1.3090 | 3.18 | 291 | ACAGGCGC-TATCGCAC | 8.8E+07 | 9.8E+07 | 1.86E+08 |  |
| **H3** | 0.954 | 49 | 3.3390 | 10.06 | 280 | CATAGAGT-TGCGGCGT | 9.8E+07 | 1.1E+08 | 2.06E+08 |  |
| **C1** | 0.635 | 47 | 2.2225 | 18.25 | 283 | TGCGAGAC-CATAATAC | 1.1E+08 | 1.2E+08 | 2.30E+08 |  |
| **C2** | 0.131 | 56 | 0.4585 | 4.52 | 299 | TCTCTACT-CGTCTGCG | 9.0E+07 | 1.0E+08 | 1.91E+08 |  |
| **C3** | 0.233 | 51 | 0.8155 | 7.89 | 281 | CTCTCGTC-TACTCATA | 1.0E+08 | 1.2E+08 | 2.19E+08 |  |

Abbreviations: cfRNA, cell-free RNA; cDNA, complementary DNA; bp, base pairs; H, healthy; C, cholestatic liver disease.

**Table S2.** Expression of enzymes, transporters and transcription factors in plasma EVs from healthy donors and donors with cholestatic liver disease.

| **Gene** | **Healthy** | **Cholestatic liver disease** | **FC** |  | **Gene** | **Healthy** | **Cholestatic liver disease** | **FC** |
| --- | --- | --- | --- | --- | --- | --- | --- | --- |
| AADAC | 0.8 ± 1.22 (3) | 1.87 ± 1.28 (3) | 2.33 |  | ALDH8A1 | 0.29 ± 0.15 (3) | 0.05 ± 0.04 (3) | 0.18 |
| ABCA1* | 0.69 ± 0.13 (3) | 2.77 ± 2.35 (3) | 4.03 |  | ALDH9A1 | 69.43 ± 19.72 (3) | 66.2 ± 29.66 (3) | 0.95 |
| ABCA10 | 0.33 ± 0.16 (3) | 0.05 ± 0.04 (3) | 0.17 |  | ALOX12B | 0.5 ± 0.25 (3) | 0.13 ± 0.15 (3) | 0.26 |
| ABCA12 | 0.49 ± 0.13 (3) | 0.06 ± 0.04 (3) | 0.12 |  | ALOX15 | 7.89 ± 6.41 (3) | 3.66 ± 3.15 (3) | 0.46 |
| ABCA13 | 0.78 ± 0.29 (3) | 0.41 ± 0.32 (3) | 0.53 |  | ALOX15B | 22.85 ± 9.62 (3) | 4.67 ± 1.51 (3) | 0.20 |
| ABCA2 | 7.03 ± 3.1 (3) | 2.49 ± 0.76 (3) | 0.35 |  | ALOX5* | 17.72 ± 9.46 (3) | 22.93 ± 11.79 (3) | 1.29 |
| ABCA3 | 1.82 ± 1.63 (3) | 17.44 ± 16.57 (3) | 9.57 |  | ALOXE3 | 0.33 ± 0.06 (3) | 0.06 ± 0.03 (3) | 0.19 |
| ABCA4 | 0.87 ± 0.34 (3) | 0.09 ± 0.08 (3) | 0.10 |  | AOC1 | 15.31 ± 2.9 (3) | 3.87 ± 0.48 (3) | 0.25 |
| ABCA5 | 3.82 ± 3.71 (3) | 1.7 ± 2.25 (3) | 0.45 |  | AOC2 | 5.4 ± 4.82 (3) | 3.09 ± 0.81 (3) | 0.57 |
| ABCA6 | 0.75 ± 0.1 (3) | 3.05 ± 2.81 (3) | 4.05 |  | AOC3* | 1.36 ± 0.64 (3) | 0.62 ± 0.57 (3) | 0.46 |
| ABCA7 | 3.72 ± 1.81 (3) | 1.19 ± 0.43 (3) | 0.32 |  | AOX1 | 0.79 ± 0.78 (3) | 0.79 ± 1.3 (3) | 1.01 |
| ABCA8 | 0.82 ± 0.17 (3) | 0.16 ± 0.12 (3) | 0.20 |  | B2M* | 2982.05 ± 392.92 (3) | 22732.45 ± 7933.04 (3) | 7.62 |
| ABCA9 | 13.99 ± 5.34 (3) | 2.96 ± 1.32 (3) | 0.21 |  | BAAT | 0.23 ± 0.05 (3) | 0.03 ± 0.03 (3) | 0.15 |
| ABCB1* | 0.94 ± 0.27 (3) | 0.18 ± 0.16 (3) | 0.19 |  | BCHE* | 0.14 ± 0.05 (2) | 0.03 (1) | - |
| ABCB10 | 24.9 ± 6.67 (3) | 23.38 ± 7.24 (3) | 0.94 |  | BLVRA | 9.97 ± 2.69 (3) | 19.47 ± 14.68 (3) | 1.95 |
| ABCB11 | 0.68 ± 0.28 (3) | 0.29 ± 0.33 (3) | 0.42 |  | BLVRB | 48.58 ± 2.09 (3) | 52.78 ± 26.84 (3) | 1.09 |
| ABCB4 | 8.59 ± 2.29 (3) | 3.18 ± 1.38 (3) | 0.37 |  | CES1 | 1.16 ± 1.06 (3) | 2.26 ± 2.7 (3) | 1.94 |
| ABCB5 | 2.37 ± 0.31 (3) | 2.82 ± 3.73 (3) | 1.19 |  | CES2 | 68.08 ± 18.32 (3) | 18.43 ± 1.91 (3) | 0.27 |
| ABCB6 | 24.9 ± 8.45 (3) | 5.89 ± 1.48 (3) | 0.24 |  | CES3 | 0.66 ± 0.71 (3) | 0.19 ± 0.31 (3) | 0.29 |
| ABCB7 | 43.26 ± 6.75 (3) | 19.1 ± 10.29 (3) | 0.44 |  | CES4A | 0.52 ± 0.5 (3) | 0.24 ± 0.27 (3) | 0.46 |
| ABCB8 | 0.99 ± 0.49 (3) | 0.12 ± 0.06 (3) | 0.12 |  | CES5A | 0.41 ± 0.14 (3) | 0.08 ± 0.06 (3) | 0.19 |
| ABCB9 | 7.24 ± 0.36 (3) | 1.71 ± 0.36 (3) | 0.24 |  | CFTR* | 0.55 ± 0.28 (3) | 0.26 ± 0.31 (3) | 0.48 |
| ABCC1* | 9.49 ± 4.74 (3) | 13.4 ± 6.04 (3) | 1.41 |  | CHDH | 4.48 ± 1.16 (3) | 2.27 ± 0.67 (3) | 0.51 |
| ABCC10 | 5.37 ± 2.64 (3) | 1.46 ± 0.6 (3) | 0.27 |  | COMT* | 9.4 ± 4.49 (3) | 23.7 ± 22.44 (3) | 2.52 |
| ABCC11 | 0.84 ± 0.38 (3) | 0.16 ± 0.05 (3) | 0.20 |  | CYP11A1* | 0.8 ± 0.27 (3) | 0.45 ± 0.33 (3) | 0.56 |
| ABCC12 | 3.03 ± 3.44 (3) | 1.24 ± 0.9 (3) | 0.41 |  | CYP11B1* | 30.34 ± 7.12 (3) | 3.31 ± 1.7 (3) | 0.11 |
| ABCC2 | 5.02 ± 2.15 (3) | 0.85 ± 0.42 (3) | 0.17 |  | CYP11B2* | 27.07 ± 12.82 (3) | 4.02 ± 2.99 (3) | 0.15 |
| ABCC3 | 1.86 ± 0.86 (3) | 23.33 ± 22.49 (3) | 12.51 |  | CYP17A1* | 10.32 ± 9.79 (3) | 2.77 ± 0.82 (3) | 0.27 |
| ABCC4 | 1.45 ± 1.02 (3) | 39.94 ± 26.07 (3) | 27.58 |  | CYP19A1* | 0.26 ± 0.05 (3) | 0.21 ± 0.24 (3) | 0.82 |
| ABCC5 | 3.42 ± 4.09 (3) | 1.18 ± 0.16 (3) | 0.35 |  | CYP1A1 | 71.37 ± 28.76 (3) | 14.45 ± 5.21 (3) | 0.20 |
| ABCC6 | 0.53 ± 0.2 (3) | 0.18 ± 0.14 (3) | 0.34 |  | CYP1A2 | 0.95 ± 0.21 (3) | 0.14 ± 0.07 (3) | 0.15 |
| ABCC8* | 3.53 ± 1.58 (3) | 1.1 ± 1.15 (3) | 0.31 |  | CYP1B1 | 11.88 ± 8.43 (3) | 4.12 ± 2.36 (3) | 0.35 |
| ABCC9 | 0.89 ± 0.38 (3) | 0.1 ± 0.03 (3) | 0.11 |  | CYP20A1 | 14.17 ± 5.41 (3) | 21.19 ± 5.43 (3) | 1.50 |
| ABCD1 | 10.39 ± 6.36 (3) | 22.03 ± 2.47 (3) | 2.12 |  | CYP21A2 | 0.33 ± 0.31 (3) | 0.05 ± 0.03 (3) | 0.15 |
| ABCD2 | 2.95 ± 4.8 (3) | 3.07 ± 2.03 (3) | 1.04 |  | CYP24A1 | 28.05 ± 10.46 (3) | 5.37 ± 2.73 (3) | 0.19 |
| ABCD3 | 23.13 ± 16.99 (3) | 22.83 ± 7.11 (3) | 0.99 |  | CYP26A1 | 0.15 ± 0.06 (3) | 0.01 ± 0.004 (2) | - |
| ABCD4 | 49.36 ± 8.68 (3) | 11.61 ± 3.92 (3) | 0.24 |  | CYP26B1 | 2.91 ± 1.56 (3) | 0.74 ± 0.32 (3) | 0.25 |
| ABCE1 | 53.86 ± 13.9 (3) | 45.9 ± 12.57 (3) | 0.85 |  | CYP26C1 | 0.2 ± 0.11 (3) | 0.02 ± 0.01 (3) | 0.10 |
| ABCF1 | 77.95 ± 22.39 (3) | 42.14 ± 25.31 (3) | 0.54 |  | CYP27A1 | 7.9 ± 10.64 (3) | 4.07 ± 2.17 (3) | 0.51 |
| ABCF2 | 42.62 ± 8.33 (3) | 30.71 ± 24.9 (3) | 0.72 |  | CYP27B1 | 0.57 ± 0.6 (3) | 0.26 ± 0.39 (3) | 0.46 |
| ABCF3 | 50.62 ± 21.05 (3) | 26.46 ± 14.18 (3) | 0.52 |  | CYP27C1 | 17.04 ± 8.03 (3) | 3.63 ± 1.86 (3) | 0.21 |
| ABCG1 | 2.72 ± 2.22 (3) | 0.72 ± 0.52 (3) | 0.26 |  | CYP2A13 | 0.28 ± 0.09 (3) | 0.05 ± 0.06 (3) | 0.18 |
| ABCG2 | 5.32 ± 1.52 (3) | 3.1 ± 2.23 (3) | 0.58 |  | CYP2A6 | 3.82 ± 2.99 (3) | 2.07 ± 0.98 (3) | 0.54 |
| ABCG4 | 27.57 ± 7.8 (3) | 6.17 ± 3.5 (3) | 0.22 |  | CYP2A7 | 3.26 ± 3.89 (3) | 1.11 ± 0.96 (3) | 0.34 |
| ABCG5 | 1.1 ± 0.39 (3) | 0.26 ± 0.08 (2) | - |  | CYP2B6 | 7.37 ± 1.88 (3) | 1.34 ± 0.6 (3) | 0.18 |
| ABCG8 | 61.65 ± 1.32 (3) | 13.56 ± 4.1 (3) | 0.22 |  | CYP2C18 | 0.09 ± 0.02 (3) | 0.03 ± 0.03 (3) | 0.41 |
| AKR1A1* | 18.98 ± 6.03 (3) | 14.28 ± 8.75 (3) | 0.75 |  | CYP2C19* | 0.79 ± 0.52 (3) | 0.62 ± 0.44 (3) | 0.79 |
| AKR1B1* | 18.77 ± 5.44 (3) | 9.82 ± 6.91 (3) | 0.52 |  | CYP2C8 | 0.13 ± 0.06 (3) | 0.02 ± 0.01 (3) | 0.12 |
| AKR1B10 | 0.45 ± 0.21 (3) | 0.38 ± 0.4 (3) | 0.84 |  | CYP2C9 | 15.49 ± 13.56 (3) | 8.58 ± 5.79 (3) | 0.55 |
| AKR1B15 | 0.21 ± 0.09 (3) | 0.02 ± 0.01 (3) | 0.10 |  | CYP2D6 | 0.24 ± 0.13 (3) | 0.09 ± 0.06 (3) | 0.37 |
| AKR1C1 | 7.02 ± 1.07 (3) | 4.38 ± 1.41 (3) | 0.62 |  | CYP2E1 | 2.06 ± 1.5 (3) | 5.86 ± 6.88 (3) | 2.84 |
| AKR1C2 | 2.19 ± 2.25 (3) | 2.35 ± 1.89 (3) | 1.07 |  | CYP2F1 | 4.95 ± 1.13 (3) | 0.88 ± 0.55 (3) | 0.18 |
| AKR1C3* | 10.44 ± 7.29 (3) | 5.06 ± 4.61 (3) | 0.49 |  | CYP2J2 | 0.32 ± 0.26 (3) | 0.2 ± 0.32 (3) | 0.65 |
| AKR1C4 | 0.14 ± 0.12 (3) | 1.11 ± 1.13 (3) | 8.06 |  | CYP2R1 | 0.38 ± 0.3 (3) | 0.88 ± 0.9 (3) | 2.31 |
| AKR1D1* | 0.27 ± 0.02 (3) | 0.07 ± 0.02 (3) | 0.28 |  | CYP2S1 | 2.53 ± 3.26 (3) | 1.1 ± 1.59 (3) | 0.43 |
| AKR1E2 | 15.31 ± 9.14 (3) | 7.54 ± 6.35 (3) | 0.49 |  | CYP2U1 | 0.64 ± 0.54 (3) | 0.94 ± 1.05 (3) | 1.45 |
| AKR7A2 | 11.46 ± 2.97 (3) | 32.92 ± 34.13 (3) | 2.87 |  | CYP2W1 | 0.38 ± 0.3 (3) | 0.1 ± 0.04 (3) | 0.27 |
| AKR7A3 | 20.03 ± 10.56 (3) | 4.3 ± 3.57 (3) | 0.21 |  | CYP39A1 | 0.29 ± 0.21 (3) | 0.17 ± 0.18 (3) | 0.59 |
| ALDH16A1 | 12.04 ± 3.39 (3) | 12.82 ± 8.36 (3) | 1.06 |  | CYP3A4* | 0.29 ± 0.2 (3) | 0.31 ± 0.26 (3) | 1.06 |
| ALDH18A1 | 6.72 ± 2.7 (3) | 4.09 ± 1.36 (3) | 0.61 |  | CYP3A43 | 10.91 ± 2.88 (3) | 2.12 ± 1.15 (3) | 0.19 |
| ALDH1A1* | 4.47 ± 3.47 (3) | 3.47 ± 2.3 (3) | 0.78 |  | CYP3A5 | 0.34 ± 0.15 (3) | 1.46 ± 1.64 (3) | 4.25 |
| ALDH1A2 | 3.46 ± 2.69 (3) | 2.45 ± 1.85 (3) | 0.71 |  | CYP3A7 | 0.4 ± 0.35 (3) | 0.01 ± 0.01 (3) | 0.03 |
| ALDH1A3 | 0.28 ± 0.08 (3) | 0.11 ± 0.05 (3) | 0.38 |  | CYP46A1 | 0.46 ± 0.19 (3) | 0.13 ± 0.1 (3) | 0.27 |
| ALDH1B1 | 1.62 ± 0.73 (3) | 0.43 ± 0.11 (3) | 0.26 |  | CYP4A11 | 0.33 ± 0.23 (3) | 0.12 ± 0.04 (3) | 0.37 |
| ALDH1L1 | 1.13 ± 0.65 (3) | 0.41 ± 0.15 (3) | 0.37 |  | CYP4A22 | 0.28 ± 0.19 (3) | 0.14 ± 0.14 (2) | - |
| ALDH1L2 | 0.41 ± 0.07 (3) | 0.09 ± 0.05 (3) | 0.21 |  | CYP4B1 | 16.94 ± 7.7 (3) | 2.39 ± 0.31 (3) | 0.14 |
| ALDH2* | 56.08 ± 5.27 (3) | 33.15 ± 4.17 (3) | 0.59 |  | CYP4F11 | 0.34 ± 0.01 (3) | 0.03 ± 0.01 (3) | 0.08 |
| ALDH3A1 | 1.17 ± 1.57 (3) | 0.46 ± 0.58 (3) | 0.39 |  | CYP4F12 | 37.09 ± 5.59 (3) | 8.58 ± 1.46 (3) | 0.23 |
| ALDH3A2 | 5.75 ± 2.44 (3) | 1.29 ± 0.94 (3) | 0.22 |  | CYP4F2 | 0.19 ± 0.07 (3) | 0.65 ± 0.52 (3) | 3.42 |
| ALDH3B1 | 17.59 ± 1.17 (3) | 20.11 ± 2.54 (3) | 1.14 |  | CYP4F22 | 0.17 ± 0.08 (3) | 0.05 ± 0.001 (2) | - |
| ALDH3B2 | 5.6 ± 5.17 (3) | 1.23 ± 0.84 (3) | 0.22 |  | CYP4F3 | 35.05 ± 14.34 (3) | 10.6 ± 6.02 (3) | 0.30 |
| ALDH4A1 | 1.3 ± 0.9 (3) | 0.74 ± 0.36 (3) | 0.57 |  | CYP4F8 | 2.63 ± 0.34 (3) | 0.46 ± 0.06 (3) | 0.18 |
| ALDH5A1* | 3.62 ± 1.3 (3) | 9.53 ± 5.88 (3) | 2.63 |  | CYP4V2 | 23.89 ± 4.14 (3) | 8.94 ± 4.97 (3) | 0.37 |
| ALDH6A1 | 1.48 ± 0.98 (3) | 0.73 ± 0.24 (3) | 0.50 |  | CYP4X1 | 0.16 ± 0.04 (3) | 1.65 ± 2.3 (2) | - |
| ALDH7A1 | 10.4 ± 13.82 (3) | 1.32 ± 0.52 (3) | 0.13 |  | CYP4Z1 | 0.29 ± 0.02 (3) | 0.98 ± 0.96 (3) | 3.36 |

**Table S2.** Continued.

| **Gene** | **Healthy** | **Cholestatic liver disease** | **FC** |  | **Gene** | **Healthy** | **Cholestatic liver disease** | **FC** |
| --- | --- | --- | --- | --- | --- | --- | --- | --- |
| CYP51A1* | 107.13 ± 45.3 (3) | 47.9 ± 13.53 (3) | 0.45 |  | MGST1 | 53.29 ± 16.14 (3) | 18.56 ± 3.84 (3) | 0.35 |
| CYP7A1 | 1.19 ± 1.76 (3) | 0.02 ± 0.01 (3) | 0.02 |  | MGST2 | 20.7 ± 5.92 (3) | 14.84 ± 5.94 (3) | 0.72 |
| CYP7B1 | 0.26 ± 0.11 (3) | 0.04 ± 0.03 (3) | 0.16 |  | MGST3 | 84.52 ± 33.74 (3) | 127.24 ± 38.24 (3) | 1.51 |
| CYP8B1 | 33.26 ± 10.17 (3) | 5.5 ± 2.19 (3) | 0.17 |  | NAT1 | 7.12 ± 1.84 (3) | 2.9 ± 2.64 (3) | 0.41 |
| DHFR* | 138.7 ± 4.41 (3) | 52.44 ± 12.57 (3) | 0.38 |  | NAT2 | 0.12 ± 0.07 (2) | 0.01 ± 0.001 (2) | - |
| DHFR2 | 0.58 ± 0.62 (3) | 0.04 ± 0.02 (3) | 0.07 |  | NDOR1 | 21.27 ± 8.31 (3) | 8.14 ± 1.59 (3) | 0.38 |
| EPHX1 | 4.89 ± 1.67 (3) | 3.16 ± 0.76 (3) | 0.65 |  | POR* | 2.05 ± 1.16 (3) | 2.79 ± 1.63 (3) | 1.36 |
| EPHX2 | 6.66 ± 4.51 (3) | 5.48 ± 7.27 (3) | 0.82 |  | SLC10A1 | 0.14 ± 0.08 (3) | 0.03 ± 0.01 (3) | 0.22 |
| EPHX3 | 0.36 ± 0.37 (3) | 0.16 ± 0.16 (3) | 0.44 |  | SLC10A2* | 0.13 ± 0.11 (3) | 0.03 ± 0.02 (3) | 0.19 |
| EPHX4 | 0.06 ± 0.04 (3) | 0.02 ± 0.02 (2) | - |  | SLC10A3 | 4.79 ± 3.37 (3) | 29.85 ± 28.64 (3) | 6.24 |
| ESD | 7.14 ± 0.44 (3) | 9.34 ± 6.51 (3) | 1.31 |  | SLC10A4 | 0.2 ± 0.09 (3) | 0.04 (1) | - |
| FCGRT | 33.03 ± 15.66 (3) | 12.8 ± 3.89 (3) | 0.39 |  | SLC10A5 | 154.58 ± 57.38 (3) | 25.73 ± 9.12 (3) | 0.17 |
| FMO1 | 0.28 ± 0.11 (3) | 0.37 ± 0.59 (3) | 1.31 |  | SLC10A6 | 0.1 ± 0.02 (3) | 0.02 ± 0.01 (3) | 0.22 |
| FMO2 | 2.58 ± 2.45 (3) | 0.72 ± 0.11 (3) | 0.28 |  | SLC10A7 | 2.26 ± 3.42 (3) | 31.13 ± 22.85 (3) | 13.78 |
| FMO3 | 1 ± 1.39 (3) | 1.7 ± 2.48 (3) | 1.70 |  | SLC11A1 | 6.09 ± 4.24 (3) | 5.38 ± 1.21 (3) | 0.88 |
| FMO4 | 0.8 ± 1.03 (3) | 0.66 ± 0.67 (3) | 0.83 |  | SLC11A2 | 7.6 ± 5.87 (3) | 6.05 ± 3.37 (3) | 0.80 |
| FMO5 | 2.67 ± 1.49 (3) | 15.4 ± 7.37 (3) | 5.77 |  | SLC12A1* | 0.61 ± 0.21 (3) | 0.04 ± 0.04 (3) | 0.06 |
| GGT1 | 14.55 ± 8.99 (3) | 5.07 ± 2.62 (3) | 0.35 |  | SLC12A2* | 2.1 ± 1 (3) | 4.02 ± 3.38 (3) | 1.91 |
| GGT5 | 1.43 ± 1.16 (3) | 0.56 ± 0.49 (3) | 0.39 |  | SLC12A3* | 2.78 ± 1.87 (3) | 0.55 ± 0.3 (3) | 0.20 |
| GGT6 | 37.92 ± 16.99 (3) | 8.05 ± 3.82 (3) | 0.21 |  | SLC12A4* | 5.69 ± 1.58 (3) | 1.51 ± 0.46 (3) | 0.26 |
| GGT7 | 1.55 ± 2.48 (3) | 0.67 ± 0.86 (2) | - |  | SLC12A5* | 0.55 ± 0.16 (3) | 0.08 ± 0.08 (3) | 0.15 |
| GLYAT | 0.18 ± 0.06 (3) | 0.33 ± 0.34 (3) | 1.81 |  | SLC12A6 | 22.35 ± 2.07 (3) | 15.57 ± 2.03 (3) | 0.70 |
| GLYATL1 | 0.02 ± 0.01 (2) | 0.02 (1) | - |  | SLC12A7 | 26.7 ± 8.11 (3) | 3.44 ± 0.9 (3) | 0.13 |
| GLYATL2 | 0.18 ± 0.09 (3) | 8.67 ± 7.14 (3) | 46.87 |  | SLC12A8 | 1.02 ± 0.42 (3) | 0.14 ± 0.03 (3) | 0.14 |
| GLYATL3 | 0.38 ± 0.16 (3) | 0.13 ± 0.08 (3) | 0.34 |  | SLC12A9 | 15.36 ± 6.38 (3) | 10.25 ± 4.9 (3) | 0.67 |
| GPX1 | 629.31 ± 202.67 (3) | 3121.67 ± 2771.83 (3) | 4.96 |  | SLC13A1 | 0.17 ± 0.07 (3) | 0.05 ± 0.03 (3) | 0.32 |
| GPX2 | 0.17 ± 0.08 (3) | 0.04 ± 0.05 (3) | 0.25 |  | SLC13A2 | 0.54 ± 0.19 (3) | 0.1 ± 0.02 (3) | 0.18 |
| GPX3 | 10.51 ± 4.92 (3) | 7.02 ± 1.09 (3) | 0.67 |  | SLC13A3 | 0.44 ± 0.15 (3) | 0.02 ± 0.02 (3) | 0.04 |
| GPX4 | 43.71 ± 3.15 (3) | 104.46 ± 53.74 (3) | 2.39 |  | SLC13A4 | 3.56 ± 1.33 (3) | 1.37 ± 0.84 (3) | 0.38 |
| GPX5 | 0.23 ± 0.11 (3) | 0.11 ± 0.02 (3) | 0.47 |  | SLC13A5 | 27.36 ± 5.89 (3) | 4.87 ± 1.03 (3) | 0.18 |
| GPX6 | 0.14 ± 0.08 (3) | 0.02 ± 0.01 (3) | 0.12 |  | SLC14A1 | 1.44 ± 0.67 (3) | 7.4 ± 6.25 (3) | 5.16 |
| GPX7 | 7.86 ± 2.97 (3) | 4.44 ± 4.23 (3) | 0.57 |  | SLC14A2 | 11.66 ± 1.18 (3) | 3.35 ± 1 (3) | 0.29 |
| GPX8 | 0.16 ± 0.09 (3) | 0.25 ± 0.24 (3) | 1.59 |  | SLC15A1 | 16.02 ± 11.19 (3) | 6.43 ± 7.4 (3) | 0.40 |
| GSTA1 | 61.12 ± 22.41 (3) | 12.87 ± 1.92 (3) | 0.21 |  | SLC15A2 | 0.51 ± 0.17 (3) | 1.23 ± 1.8 (3) | 2.41 |
| GSTA2 | 0.11 ± 0.04 (3) | 0.59 ± 0.74 (3) | 5.24 |  | SLC15A3 | 17.9 ± 6.74 (3) | 13.78 ± 9.81 (3) | 0.77 |
| GSTA3 | 0.2 ± 0.08 (3) | 0.02 ± 0.02 (3) | 0.12 |  | SLC15A4 | 14.61 ± 12.47 (3) | 6.61 ± 1.73 (3) | 0.45 |
| GSTA4 | 2.46 ± 2.91 (3) | 4.56 ± 3.46 (3) | 1.86 |  | SLC15A5 | 0.16 ± 0.11 (3) | 0.03 ± 0.003 (3) | 0.17 |
| GSTA5 | 0.36 ± 0.18 (3) | 0.35 ± 0.51 (3) | 0.97 |  | SLC16A1* | 17.72 ± 2.24 (3) | 10.02 ± 3.62 (3) | 0.57 |
| GSTK1 | 215 ± 46.83 (3) | 180.55 ± 22.86 (3) | 0.84 |  | SLC16A10 | 0.83 ± 0.48 (3) | 0.34 ± 0.32 (3) | 0.42 |
| GSTM1 | 8.08 ± 1.75 (3) | 11.36 ± 19.3 (3) | 1.41 |  | SLC16A11 | 49.52 ± 10.56 (3) | 9.38 ± 3.92 (3) | 0.19 |
| GSTM2 | 0.26 ± 0.11 (3) | 0.01 ± 0.01 (3) | 0.06 |  | SLC16A12 | 26.18 ± 5.4 (3) | 7.51 ± 2.87 (3) | 0.29 |
| GSTM3 | 9.25 ± 5.15 (3) | 9.21 ± 9.09 (3) | 1.00 |  | SLC16A13 | 3.97 ± 4.88 (3) | 1.91 ± 1.64 (3) | 0.48 |
| GSTM4 | 0.78 ± 0.6 (3) | 1.67 ± 1.95 (3) | 2.13 |  | SLC16A14 | 1.91 ± 2.3 (3) | 0.29 ± 0.49 (3) | 0.15 |
| GSTM5 | 0.83 ± 0.55 (3) | 8.87 ± 6.27 (3) | 10.75 |  | SLC16A2 | 0.71 ± 0.14 (3) | 0.21 ± 0.2 (3) | 0.30 |
| GSTO1 | 20.37 ± 3.85 (3) | 13.8 ± 7.27 (3) | 0.68 |  | SLC16A3 | 34.67 ± 35.19 (3) | 17.91 ± 9.91 (3) | 0.52 |
| GSTO2 | 1.19 ± 0.35 (3) | 0.67 ± 0.82 (3) | 0.56 |  | SLC16A4 | 11.75 ± 4.19 (3) | 2.71 ± 0.47 (3) | 0.23 |
| GSTP1 | 181.27 ± 34.08 (3) | 114.92 ± 52.09 (3) | 0.63 |  | SLC16A5 | 0.38 ± 0.29 (3) | 0.97 ± 0.46 (3) | 2.54 |
| GSTT2 | 0.04 (1) | 0.03 (1) | - |  | SLC16A6 | 3.03 ± 3.14 (3) | 3.21 ± 0.86 (3) | 1.06 |
| GSTT2B | 0.04 (1) | 0.01 ± 0.003 (2) | - |  | SLC16A7 | 0.36 ± 0.02 (3) | 0.06 ± 0.02 (3) | 0.17 |
| GSTT4 | 1.16 ± 0.87 (3) | 0.42 ± 0.51 (3) | 0.36 |  | SLC16A8 | 3.86 ± 1.9 (3) | 0.71 ± 0.5 (3) | 0.18 |
| GSTZ1 | 2.08 ± 0.98 (3) | 0.95 ± 0.46 (3) | 0.46 |  | SLC16A9 | 0.14 ± 0.06 (3) | 11.67 ± 19.81 (3) | 85.05 |
| LOXL2* | 0.91 ± 0.44 (3) | 0.21 ± 0.15 (3) | 0.24 |  | SLC17A1 | 9.93 ± 5.14 (3) | 2.62 ± 2.13 (3) | 0.26 |
| LOXL3 | 1.36 ± 1.23 (3) | 8.44 ± 9.48 (3) | 6.21 |  | SLC17A2 | 0.26 ± 0.11 (3) | 0.02 ± 0.01 (3) | 0.08 |
| LOXL4 | 0.27 ± 0.06 (3) | 0.11 ± 0.16 (3) | 0.41 |  | SLC17A3 | 16.27 ± 4.67 (3) | 5.58 ± 2.26 (3) | 0.34 |
| LTA4H | 90.26 ± 4.63 (3) | 88.38 ± 19.11 (3) | 0.98 |  | SLC17A4 | 0.42 ± 0.23 (3) | 0.34 ± 0.45 (3) | 0.81 |
| MAOA* | 2.04 ± 1.01 (3) | 3.27 ± 1.28 (3) | 1.60 |  | SLC17A5 | 8.78 ± 7.45 (3) | 10.14 ± 5.03 (3) | 1.16 |
| MAOB* | 0.85 ± 1.08 (3) | 19.68 ± 19.01 (3) | 23.2 |  | SLC17A6 | 0.25 ± 0.14 (3) | 0.01 ± 0.01 (3) | 0.06 |
| MFSD1 | 16.89 ± 10.78 (3) | 200.53 ± 176.21 (3) | 11.88 |  | SLC17A7 | 0.51 ± 0.39 (3) | 0.2 ± 0.15 (3) | 0.40 |
| MFSD10 | 4.51 ± 2.33 (3) | 4.89 ± 1.82 (3) | 1.08 |  | SLC17A8 | 0.6 ± 0.38 (3) | 0.31 ± 0.49 (3) | 0.52 |
| MFSD11 | 15.41 ± 8.91 (3) | 19.92 ± 6.08 (3) | 1.29 |  | SLC17A9 | 0.19 ± 0.09 (3) | 0.14 ± 0.1 (3) | 0.73 |
| MFSD12 | 1.48 ± 0.34 (3) | 0.67 ± 0.17 (3) | 0.45 |  | SLC18A1* | 0.87 ± 0.1 (3) | 0.18 ± 0.12 (3) | 0.21 |
| MFSD13A | 49.02 ± 17.94 (3) | 8.34 ± 2.52 (3) | 0.17 |  | SLC18A2* | 21.31 ± 18.08 (3) | 48.48 ± 44.92 (3) | 2.28 |
| MFSD14A | 1.9 ± 1.39 (3) | 3.56 ± 0.51 (3) | 1.87 |  | SLC18A3 | 98.25 ± 38.33 (3) | 13.14 ± 8.03 (3) | 0.13 |
| MFSD14B | 17.94 ± 9.79 (3) | 19.23 ± 0.79 (3) | 1.07 |  | SLC18B1 | 2.84 ± 2.15 (3) | 10.84 ± 7.53 (3) | 3.82 |
| MFSD14C | 9.01 ± 5.39 (3) | 4.54 ± 1.64 (3) | 0.50 |  | SLC19A1 | 1.02 ± 0.31 (3) | 0.75 ± 0.66 (3) | 0.74 |
| MFSD2A | 0.49 ± 0.45 (3) | 0.45 ± 0.21 (3) | 0.91 |  | SLC19A2 | 0.17 ± 0.15 (3) | 0.33 ± 0.33 (3) | 1.93 |
| MFSD2B | 0.98 ± 0.77 (3) | 18.04 ± 12.69 (3) | 18.5 |  | SLC19A3 | 6.09 ± 1.69 (3) | 1.28 ± 0.08 (3) | 0.21 |
| MFSD3 | 82.01 ± 24.55 (3) | 14.57 ± 5.52 (3) | 0.18 |  | SLC1A1* | 2.35 ± 1.36 (3) | 0.88 ± 0.27 (3) | 0.38 |
| MFSD4A | 99.09 ± 14.37 (3) | 18.09 ± 4.73 (3) | 0.18 |  | SLC1A2* | 12.57 ± 5.19 (3) | 1.69 ± 0.45 (3) | 0.13 |
| MFSD4B | 51.92 ± 28.44 (3) | 387.84 ± 301.76 (3) | 7.47 |  | SLC1A3* | 0.89 ± 0.19 (3) | 0.29 ± 0.14 (3) | 0.33 |
| MFSD5 | 0.25 ± 0.12 (3) | 0.02 ± 0.004 (2) | - |  | SLC1A4* | 6.87 ± 10.61 (3) | 24.89 ± 27.43 (3) | 3.62 |
| MFSD6 | 2 ± 1.39 (3) | 8.81 ± 4.99 (3) | 4.40 |  | SLC1A5 | 20.96 ± 12.4 (3) | 19.99 ± 8.53 (3) | 0.95 |
| MFSD6L | 452.84 ± 186.56 (3) | 77.83 ± 42.03 (3) | 0.17 |  | SLC1A6* | 6.9 ± 1.23 (3) | 1.64 ± 0.81 (3) | 0.24 |
| MFSD8 | 8.68 ± 3.74 (3) | 2.59 ± 2.4 (3) | 0.30 |  | SLC1A7* | 0.82 ± 0.75 (3) | 0.17 ± 0.15 (3) | 0.20 |
| MFSD9 | 1.72 ± 0.97 (3) | 2.85 ± 0.54 (3) | 1.65 |  | SLC20A1 | 63.55 ± 21.87 (3) | 25.82 ± 6.71 (3) | 0.41 |

**Table S2.** Continued.

| **Gene** | **Healthy** | **Cholestatic liver disease** | **FC** |  | **Gene** | **Healthy** | **Cholestatic liver disease** | **FC** |
| --- | --- | --- | --- | --- | --- | --- | --- | --- |
| SLC20A2 | 1.09 ± 0.87 (3) | 3.08 ± 1.74 (3) | 2.82 |  | SLC25A51 | 20.42 ± 11.39 (3) | 9.31 ± 3 (3) | 0.46 |
| SLC22A1 | 0.27 ± 0.14 (3) | 0.3 ± 0.26 (3) | 1.14 |  | SLC25A52 | 167.64 ± 58 (3) | 27.09 ± 13.06 (3) | 0.16 |
| SLC22A10 | 0.75 ± 0.44 (3) | 0.48 ± 0.46 (3) | 0.64 |  | SLC25A53 | 0.39 ± 0.11 (3) | 0.1 ± 0.06 (3) | 0.26 |
| SLC22A11* | 0.58 ± 0.54 (3) | 0.08 ± 0.06 (3) | 0.15 |  | SLC25A6* | 323.13 ± 94.86 (3) | 201.86 ± 154.04 (3) | 0.62 |
| SLC22A12* | 0.28 ± 0.27 (3) | 0.03 ± 0.01 (2) | - |  | SLC26A1 | 0.19 ± 0.1 (3) | 0.02 ± 0.01 (2) | - |
| SLC22A13 | 29.92 ± 7.73 (3) | 7.29 ± 3.62 (3) | 0.24 |  | SLC26A11 | 3.99 ± 2.41 (3) | 1.93 ± 1.55 (3) | 0.48 |
| SLC22A14 | 13.95 ± 10.25 (3) | 3.09 ± 1.33 (3) | 0.22 |  | SLC26A2 | 1.51 ± 1.64 (3) | 1.48 ± 1.56 (3) | 0.98 |
| SLC22A15 | 1.03 ± 1.47 (3) | 1.07 ± 0.84 (3) | 1.03 |  | SLC26A3 | 0.75 ± 0.5 (3) | 0.31 ± 0.18 (3) | 0.41 |
| SLC22A16 | 2.73 ± 2.89 (3) | 2.19 ± 1.34 (3) | 0.80 |  | SLC26A4 | 2.35 ± 0.75 (3) | 1.16 ± 0.98 (3) | 0.49 |
| SLC22A17 | 10.81 ± 9.34 (3) | 5.64 ± 1.05 (3) | 0.52 |  | SLC26A5 | 0.18 ± 0.07 (3) | 0.01 ± 0.01 (2) | - |
| SLC22A18 | 5.84 ± 1.32 (3) | 1.06 ± 0.21 (3) | 0.18 |  | SLC26A6 | 44.01 ± 11.08 (3) | 9.27 ± 2.03 (3) | 0.21 |
| SLC22A2 | 0.3 ± 0.15 (3) | 0.04 ± 0.01 (3) | 0.12 |  | SLC26A7 | 0.13 ± 0.08 (3) | 0.23 ± 0.31 (2) | - |
| SLC22A23 | 2.18 ± 2.02 (3) | 8.93 ± 3.27 (3) | 4.09 |  | SLC26A8 | 1.79 ± 1.79 (3) | 3.01 ± 3.26 (3) | 1.68 |
| SLC22A24 | 0.11 ± 0.06 (3) | 0.03 ± 0.001 (2) | - |  | SLC26A9 | 0.66 ± 0.17 (3) | 0.03 ± 0.02 (3) | 0.05 |
| SLC22A25 | 14.6 ± 9.81 (3) | 4.47 ± 2.41 (3) | 0.31 |  | SLC27A1 | 12.49 ± 3.03 (3) | 2.85 ± 1.68 (3) | 0.23 |
| SLC22A3 | 0.27 ± 0.15 (3) | 0.03 ± 0.01 (3) | 0.09 |  | SLC27A2 | 1.11 ± 1.74 (3) | 0.09 ± 0.1 (3) | 0.08 |
| SLC22A31 | 44.07 ± 16.27 (3) | 12.46 ± 6.38 (3) | 0.28 |  | SLC27A3 | 2.52 ± 2.02 (3) | 3.37 ± 2.6 (3) | 1.34 |
| SLC22A4 | 0.9 ± 0.64 (3) | 0.59 ± 0.14 (3) | 0.66 |  | SLC27A4 | 0.36 ± 0.06 (3) | 0.23 ± 0.05 (3) | 0.62 |
| SLC22A5 | 3.58 ± 1.22 (3) | 0.53 ± 0.49 (3) | 0.15 |  | SLC27A5 | 76.85 ± 21.51 (3) | 19.49 ± 6.98 (3) | 0.25 |
| SLC22A6* | 4.47 ± 3.41 (3) | 0.85 ± 0.82 (3) | 0.19 |  | SLC27A6 | 7.84 ± 7.21 (3) | 3.16 ± 2.95 (3) | 0.40 |
| SLC22A7 | 18.43 ± 8.87 (3) | 4.32 ± 1.74 (3) | 0.23 |  | SLC28A1 | 0.56 ± 0.26 (3) | 0.12 ± 0.01 (3) | 0.22 |
| SLC22A8* | 1.61 ± 1.15 (3) | 0.41 ± 0.45 (2) | - |  | SLC28A2 | 1.7 ± 0.89 (3) | 0.72 ± 0.71 (3) | 0.42 |
| SLC22A9 | 0.81 ± 0.24 (3) | 0.21 ± 0.09 (3) | 0.26 |  | SLC28A3 | 0.69 ± 0.41 (3) | 0.29 ± 0.29 (3) | 0.42 |
| SLC23A1 | 7.18 ± 5.88 (3) | 2.13 ± 1.31 (3) | 0.30 |  | SLC29A1 | 3.6 ± 3.74 (3) | 8.54 ± 7.61 (3) | 2.37 |
| SLC23A2 | 0.65 ± 0.37 (3) | 3.79 ± 2.27 (3) | 5.83 |  | SLC29A2 | 5.91 ± 4.41 (3) | 1.53 ± 1.02 (3) | 0.26 |
| SLC23A3 | 0.56 ± 0.37 (3) | 0.36 ± 0.31 (3) | 0.65 |  | SLC29A3 | 0.45 ± 0.09 (3) | 0.72 ± 0.67 (3) | 1.61 |
| SLC24A1 | 9.07 ± 5.45 (3) | 3.85 ± 2.29 (3) | 0.43 |  | SLC29A4 | 39.73 ± 8.94 (3) | 8.88 ± 4.26 (3) | 0.22 |
| SLC24A2 | 0.62 ± 0.34 (3) | 0.11 ± 0.03 (2) | - |  | SLC2A1 | 649.79 ± 622.37 (3) | 233.34 ± 134.84 (3) | 0.36 |
| SLC24A3 | 3.75 ± 5.69 (3) | 82.26 ± 83.96 (3) | 21.92 |  | SLC2A10 | 0.53 ± 0.29 (3) | 0.09 ± 0.06 (3) | 0.17 |
| SLC24A4 | 109.22 ± 21.48 (3) | 24.33 ± 7.77 (3) | 0.22 |  | SLC2A11 | 6.23 ± 2.85 (3) | 8.79 ± 3.21 (3) | 1.41 |
| SLC24A5 | 0.15 ± 0.11 (3) | 0.24 ± 0.24 (3) | 1.57 |  | SLC2A12 | 0.19 ± 0.09 (3) | 0.02 ± 0.01 (3) | 0.11 |
| SLC25A1 | 37.95 ± 4.74 (3) | 25.25 ± 6.99 (3) | 0.67 |  | SLC2A13 | 0.75 ± 0.66 (3) | 3.22 ± 2.43 (3) | 4.29 |
| SLC25A10 | 0.83 ± 1.16 (3) | 0.62 ± 0.29 (3) | 0.75 |  | SLC2A14 | 1.41 ± 1.36 (3) | 0.17 ± 0.06 (3) | 0.12 |
| SLC25A11 | 71.61 ± 13.47 (3) | 55.5 ± 31.03 (3) | 0.77 |  | SLC2A2 | 0.23 ± 0.09 (3) | 0.02 ± 0.02 (2) | - |
| SLC25A12 | 0.8 ± 0.26 (3) | 1.83 ± 1.5 (3) | 2.28 |  | SLC2A3 | 59.07 ± 23.73 (3) | 311.53 ± 196.49 (3) | 5.27 |
| SLC25A13 | 0.19 ± 0.06 (3) | 0.89 ± 0.73 (2) | - |  | SLC2A4 | 0.68 ± 0.43 (3) | 1.03 ± 0.58 (3) | 1.52 |
| SLC25A14 | 0.36 ± 0.18 (3) | 0.12 ± 0.08 (3) | 0.34 |  | SLC2A5 | 18.52 ± 8.08 (3) | 5.64 ± 1.46 (3) | 0.30 |
| SLC25A15 | 34.02 ± 12.59 (3) | 13.16 ± 6.58 (3) | 0.39 |  | SLC2A6 | 17.28 ± 7.9 (3) | 3.95 ± 0.43 (3) | 0.23 |
| SLC25A16 | 30.79 ± 16.43 (3) | 81.76 ± 33.54 (3) | 2.66 |  | SLC2A7 | 0.17 ± 0.07 (3) | 0.03 ± 0.03 (2) | - |
| SLC25A17 | 13.28 ± 4.23 (3) | 18.99 ± 12.73 (3) | 1.43 |  | SLC2A8 | 0.69 ± 0.31 (3) | 0.47 ± 0.27 (3) | 0.68 |
| SLC25A18 | 1.91 ± 1.42 (3) | 0.73 ± 0.62 (3) | 0.38 |  | SLC2A9 | 1.18 ± 0.84 (3) | 0.49 ± 0.59 (3) | 0.42 |
| SLC25A19 | 5.6 ± 2.24 (3) | 8.35 ± 4.86 (3) | 1.49 |  | SLC30A1 | 22.24 ± 13.9 (3) | 17.44 ± 6.75 (3) | 0.78 |
| SLC25A2 | 88.13 ± 30.82 (3) | 15.94 ± 8.75 (3) | 0.18 |  | SLC30A10 | 4.66 ± 1.06 (3) | 1.23 ± 0.3 (3) | 0.26 |
| SLC25A20 | 10.87 ± 3.32 (3) | 8.32 ± 3.2 (3) | 0.77 |  | SLC30A2 | 0.79 ± 0.62 (3) | 0.07 ± 0.01 (3) | 0.09 |
| SLC25A21 | 0.88 ± 1.31 (3) | 0.19 ± 0.24 (2) | - |  | SLC30A3 | 0.44 ± 0.23 (3) | 0.47 ± 0.66 (3) | 1.05 |
| SLC25A22 | 6.52 ± 1.85 (3) | 4.65 ± 0.98 (3) | 0.71 |  | SLC30A4 | 17.48 ± 4.37 (3) | 8.43 ± 3.89 (3) | 0.48 |
| SLC25A23 | 16.86 ± 6.58 (3) | 10.52 ± 5.49 (3) | 0.62 |  | SLC30A5 | 3.3 ± 1.97 (3) | 13.95 ± 5.89 (3) | 4.23 |
| SLC25A24 | 16.81 ± 7.69 (3) | 16.45 ± 8.15 (3) | 0.98 |  | SLC30A6 | 0.59 ± 0.06 (3) | 0.24 ± 0.09 (3) | 0.42 |
| SLC25A25 | 5.23 ± 2.67 (3) | 6.66 ± 5.24 (3) | 1.27 |  | SLC30A7 | 13.11 ± 4.16 (3) | 63.58 ± 23.29 (3) | 4.85 |
| SLC25A26 | 4.72 ± 4.45 (3) | 2.95 ± 1.81 (3) | 0.63 |  | SLC30A8 | 0.39 ± 0.07 (3) | 0.06 ± 0.04 (3) | 0.15 |
| SLC25A27 | 0.19 ± 0.06 (3) | 0.03 ± 0.01 (3) | 0.15 |  | SLC30A9 | 20.27 ± 5.36 (3) | 30.19 ± 9.24 (3) | 1.49 |
| SLC25A28 | 7.85 ± 5.37 (3) | 3.01 ± 1.09 (3) | 0.38 |  | SLC31A1 | 3.63 ± 1.65 (3) | 18.19 ± 11.17 (3) | 5.01 |
| SLC25A29 | 7.48 ± 3.45 (3) | 1.87 ± 1.07 (3) | 0.25 |  | SLC31A2 | 0.11 ± 0.04 (3) | 0.23 ± 0.27 (2) | - |
| SLC25A3 | 93.8 ± 33.97 (3) | 64.8 ± 39.95 (3) | 0.69 |  | SLC32A1* | 0.13 ± 0.08 (3) | 0.02 ± 0.01 (3) | 0.13 |
| SLC25A30 | 115.75 ± 22.31 (3) | 38.76 ± 4.43 (3) | 0.33 |  | SLC33A1 | 5.06 ± 3.32 (3) | 13.38 ± 1.02 (3) | 2.64 |
| SLC25A31 | 0.07 ± 0.03 (3) | 0.04 (1) | - |  | SLC34A1 | 4.58 ± 3.32 (3) | 1.88 ± 1.66 (3) | 0.41 |
| SLC25A32 | 1.85 ± 1.69 (3) | 0.52 ± 0.34 (3) | 0.28 |  | SLC34A2* | 0.52 ± 0.21 (3) | 0.07 ± 0.07 (3) | 0.13 |
| SLC25A33 | 10.21 ± 1.12 (3) | 3.47 ± 1.61 (3) | 0.34 |  | SLC34A3 | 19.29 ± 11.82 (3) | 5.05 ± 1.87 (3) | 0.26 |
| SLC25A34 | 0.27 ± 0.06 (3) | 0.05 ± 0.02 (3) | 0.18 |  | SLC35A1 | 4.44 ± 7.47 (3) | 5.6 ± 4.74 (3) | 1.26 |
| SLC25A35 | 10.22 ± 10.2 (3) | 0.9 ± 0.84 (3) | 0.09 |  | SLC35A2 | 1.14 ± 0.42 (3) | 0.56 ± 0.6 (3) | 0.49 |
| SLC25A36 | 26.46 ± 6.89 (3) | 20.81 ± 10.7 (3) | 0.79 |  | SLC35A3 | 16.32 ± 7.67 (3) | 19.76 ± 11.08 (3) | 1.21 |
| SLC25A37 | 129.06 ± 74.89 (3) | 369.13 ± 283.44 (3) | 2.86 |  | SLC35A4 | 60.66 ± 20.12 (3) | 57.27 ± 34.16 (3) | 0.94 |
| SLC25A38 | 7.71 ± 1.31 (3) | 7.64 ± 7.28 (3) | 0.99 |  | SLC35A5 | 114.09 ± 112.27 (3) | 62.28 ± 31.96 (3) | 0.55 |
| SLC25A39 | 124.48 ± 34.12 (3) | 159.68 ± 77.42 (3) | 1.28 |  | SLC35B1 | 9.23 ± 8.07 (3) | 9.27 ± 1.45 (3) | 1.00 |
| SLC25A4* | 10.23 ± 0.76 (3) | 6.9 ± 4.26 (3) | 0.67 |  | SLC35B2 | 2.26 ± 1.06 (3) | 0.9 ± 0.39 (3) | 0.40 |
| SLC25A40 | 6.3 ± 2.11 (3) | 8.61 ± 2.89 (3) | 1.37 |  | SLC35B3 | 5.64 ± 3.25 (3) | 4.15 ± 2.64 (3) | 0.74 |
| SLC25A41 | 0.64 ± 0.33 (3) | 0.12 ± 0.07 (3) | 0.19 |  | SLC35B4 | 55.28 ± 14.6 (3) | 13.13 ± 4.2 (3) | 0.24 |
| SLC25A42 | 2.09 ± 1.08 (3) | 3.31 ± 2.88 (3) | 1.58 |  | SLC35C1 | 4.21 ± 1.04 (3) | 2.94 ± 2.11 (3) | 0.70 |
| SLC25A43 | 3.52 ± 0.99 (3) | 3.99 ± 3.51 (3) | 1.13 |  | SLC35C2 | 28.04 ± 4.31 (3) | 32.05 ± 16.62 (3) | 1.14 |
| SLC25A44 | 34.35 ± 4.9 (3) | 22.79 ± 11.92 (3) | 0.66 |  | SLC35D1 | 4.43 ± 3.33 (3) | 7.07 ± 4.33 (3) | 1.60 |
| SLC25A45 | 81.98 ± 9.82 (3) | 20.24 ± 3.74 (3) | 0.25 |  | SLC35D2 | 4.4 ± 1.94 (3) | 39.52 ± 26.62 (3) | 8.99 |
| SLC25A46 | 10.12 ± 2.15 (3) | 14.83 ± 6.44 (3) | 1.47 |  | SLC35D3 | 0.62 ± 0.3 (3) | 0.21 ± 0.15 (3) | 0.34 |
| SLC25A47 | 0.5 ± 0.47 (3) | 0.35 ± 0.27 (3) | 0.70 |  | SLC35E1 | 12.95 ± 3.46 (3) | 57.69 ± 25.48 (3) | 4.45 |
| SLC25A48 | 0.73 ± 0.35 (3) | 0.03 ± 0.01 (3) | 0.05 |  | SLC35E3 | 25.41 ± 4.51 (3) | 6.89 ± 0.31 (3) | 0.27 |
| SLC25A5* | 236.5 ± 7.59 (3) | 227.27 ± 115.89 (3) | 0.96 |  | SLC35E4 | 1.3 ± 0.2 (3) | 0.21 ± 0.14 (3) | 0.16 |

**Table S2.** Continued.

| **Gene** | **Healthy** | **Cholestatic liver disease** | **FC** |  | **Gene** | **Healthy** | **Cholestatic liver disease** | **FC** |
| --- | --- | --- | --- | --- | --- | --- | --- | --- |
| SLC35F1 | 0.31 ± 0.17 (3) | 0.07 ± 0.05 (3) | 0.22 |  | SLC4A4 | 0.54 ± 0.42 (3) | 2 ± 2.36 (3) | 3.70 |
| SLC35F2 | 6.71 ± 2.85 (3) | 4.37 ± 2.48 (3) | 0.65 |  | SLC4A5 | 0.57 ± 0.1 (3) | 0.17 ± 0.1 (3) | 0.30 |
| SLC35F3 | 1.54 ± 0.63 (3) | 0.2 ± 0.19 (3) | 0.13 |  | SLC4A7 | 13.44 ± 11.89 (3) | 6.78 ± 4.93 (3) | 0.50 |
| SLC35F4 | 0.27 ± 0.24 (3) | 0.03 ± 0.02 (3) | 0.11 |  | SLC4A8 | 7.46 ± 1.33 (3) | 1.33 ± 0.26 (3) | 0.18 |
| SLC35F5 | 3.57 ± 2.52 (3) | 60.82 ± 48.56 (3) | 17.05 |  | SLC4A9 | 18.64 ± 6.92 (3) | 4.45 ± 2.71 (3) | 0.24 |
| SLC35F6 | 12.47 ± 4.73 (3) | 4.12 ± 2.59 (3) | 0.33 |  | SLC50A1 | 127.12 ± 38.98 (3) | 139.56 ± 84.63 (3) | 1.10 |
| SLC35G1 | 0.45 ± 0.24 (3) | 0.17 ± 0.28 (3) | 0.38 |  | SLC51A | 11.01 ± 3.41 (3) | 1.94 ± 0.13 (3) | 0.18 |
| SLC35G2 | 2.49 ± 0.43 (3) | 0.49 ± 0.17 (3) | 0.20 |  | SLC51B | 0.78 ± 1.05 (3) | 0.05 ± 0.03 (3) | 0.06 |
| SLC35G3 | 242.18 ± 102.2 (3) | 36.34 ± 16.35 (3) | 0.15 |  | SLC52A1 | 27.31 ± 14.33 (3) | 5.75 ± 2.8 (3) | 0.21 |
| SLC35G5 | 255.94 ± 78.09 (3) | 38.26 ± 14.54 (3) | 0.15 |  | SLC52A2* | 35.46 ± 14.55 (3) | 14.72 ± 8.04 (3) | 0.42 |
| SLC35G6 | 167.92 ± 56.67 (3) | 35.85 ± 15.35 (3) | 0.21 |  | SLC52A3 | 13.1 ± 3.91 (3) | 3.08 ± 0.53 (3) | 0.23 |
| SLC36A1 | 2.53 ± 1.87 (3) | 1.96 ± 1.22 (3) | 0.77 |  | SLC5A1 | 0.76 ± 0.19 (3) | 0.12 ± 0.05 (3) | 0.16 |
| SLC36A2 | 0.42 ± 0.06 (3) | 0.09 ± 0.03 (3) | 0.21 |  | SLC5A10 | 1.34 ± 0.89 (3) | 0.16 ± 0.08 (3) | 0.12 |
| SLC36A3 | 0.38 ± 0.1 (3) | 0.05 ± 0.02 (3) | 0.13 |  | SLC5A11 | 6.36 ± 3.07 (3) | 2.11 ± 1.74 (3) | 0.33 |
| SLC36A4 | 9.55 ± 5.97 (3) | 13.25 ± 0.29 (3) | 1.39 |  | SLC5A12 | 0.35 ± 0.13 (3) | 0.15 ± 0.22 (3) | 0.43 |
| SLC37A1 | 0.31 ± 0.08 (3) | 0.06 ± 0.02 (3) | 0.19 |  | SLC5A2* | 50.73 ± 28.2 (3) | 10.7 ± 5.21 (3) | 0.21 |
| SLC37A2 | 2.03 ± 0.98 (3) | 1.51 ± 1.33 (3) | 0.74 |  | SLC5A3 | 0.28 ± 0.11 (3) | 0.07 ± 0.03 (3) | 0.26 |
| SLC37A3 | 7.67 ± 3 (3) | 5.33 ± 2.55 (3) | 0.70 |  | SLC5A4 | 2.19 ± 2.64 (3) | 0.29 ± 0.19 (3) | 0.13 |
| SLC37A4 | 0.28 ± 0.18 (3) | 0.05 ± 0.04 (3) | 0.18 |  | SLC5A5 | 0.79 ± 0.46 (3) | 0.13 ± 0.08 (3) | 0.16 |
| SLC38A1 | 4.46 ± 3.81 (3) | 4.34 ± 4.03 (3) | 0.97 |  | SLC5A6 | 10.28 ± 6.83 (3) | 9.08 ± 9.21 (3) | 0.88 |
| SLC38A10 | 2.28 ± 0.87 (3) | 4.41 ± 1.61 (3) | 1.93 |  | SLC5A7 | 0.19 ± 0.01 (3) | 0.05 ± 0.03 (2) | - |
| SLC38A11 | 0.85 ± 0.67 (3) | 2.81 ± 2.12 (2) | - |  | SLC5A8 | 0.31 ± 0.06 (3) | 0.06 ± 0.05 (3) | 0.20 |
| SLC38A2 | 26.01 ± 5.72 (3) | 36.59 ± 19.51 (3) | 1.41 |  | SLC5A9 | 0.83 ± 0.63 (3) | 0.07 ± 0.03 (3) | 0.08 |
| SLC38A3 | 45.91 ± 22.15 (3) | 9.58 ± 5.84 (3) | 0.21 |  | SLC66A1 | 3.29 ± 3.04 (3) | 6.05 ± 2.27 (3) | 1.84 |
| SLC38A4 | 0.3 ± 0.16 (3) | 0.49 ± 0.57 (3) | 1.63 |  | SLC66A2 | 4.38 ± 0.88 (3) | 4.86 ± 0.7 (3) | 1.11 |
| SLC38A5 | 30.24 ± 8.74 (3) | 8.41 ± 2.64 (3) | 0.28 |  | SLC66A3 | 45.8 ± 18.17 (3) | 17.46 ± 0.54 (3) | 0.38 |
| SLC38A6 | 3.13 ± 1.44 (3) | 5.25 ± 5.49 (3) | 1.68 |  | SLC6A1* | 17.86 ± 4.37 (3) | 2.82 ± 0.49 (3) | 0.16 |
| SLC38A7 | 15.14 ± 5.65 (3) | 2.66 ± 0.11 (3) | 0.18 |  | SLC6A11* | 0.57 ± 0.21 (3) | 0.07 ± 0.07 (3) | 0.11 |
| SLC38A8 | 0.18 ± 0.18 (3) | 0.02 ± 0.01 (3) | 0.13 |  | SLC6A12* | 3.98 ± 1.2 (3) | 0.57 ± 0.23 (3) | 0.14 |
| SLC38A9 | 1.57 ± 1.6 (3) | 6.77 ± 3.67 (3) | 4.31 |  | SLC6A13* | 0.58 ± 0.25 (3) | 0.05 ± 0.03 (3) | 0.08 |
| SLC39A1 | 17.52 ± 8.05 (3) | 12.84 ± 5.94 (3) | 0.73 |  | SLC6A14 | 0.16 ± 0.16 (3) | 0.06 ± 0.03 (2) | - |
| SLC39A10 | 0.14 ± 0.06 (3) | 0.17 ± 0.13 (3) | 1.25 |  | SLC6A15 | 4.89 ± 2.09 (3) | 1.54 ± 0.77 (3) | 0.32 |
| SLC39A11 | 0.56 ± 0.45 (3) | 0.49 ± 0.42 (3) | 0.88 |  | SLC6A16 | 16.67 ± 8.28 (3) | 4.95 ± 1.94 (3) | 0.30 |
| SLC39A12 | 0.29 ± 0.1 (3) | 0.07 ± 0.01 (3) | 0.24 |  | SLC6A17 | 0.64 ± 0.25 (3) | 0.06 ± 0.01 (3) | 0.10 |
| SLC39A13 | 4.67 ± 2.48 (3) | 1.21 ± 0.33 (3) | 0.26 |  | SLC6A18 | 0.39 ± 0.26 (3) | 0.13 ± 0.15 (3) | 0.33 |
| SLC39A14 | 2.63 ± 0.73 (3) | 0.63 ± 0.24 (3) | 0.24 |  | SLC6A19 | 108.33 ± 21.09 (3) | 16.88 ± 10.69 (3) | 0.16 |
| SLC39A2 | 0.16 ± 0.09 (3) | 0.02 ± 0.01 (3) | 0.12 |  | SLC6A2* | 1.02 ± 0.53 (3) | 0.15 ± 0.03 (3) | 0.15 |
| SLC39A3 | 4.64 ± 1.72 (3) | 29.86 ± 20.3 (3) | 6.44 |  | SLC6A20 | 3.12 ± 1.44 (3) | 0.46 ± 0.3 (3) | 0.15 |
| SLC39A4 | 23.45 ± 7.05 (3) | 13.94 ± 3.83 (3) | 0.59 |  | SLC6A3* | 0.6 ± 0.17 (3) | 0.07 ± 0.04 (3) | 0.12 |
| SLC39A5 | 8.04 ± 6.56 (3) | 3.58 ± 1.1 (3) | 0.44 |  | SLC6A4* | 6.12 ± 2.96 (3) | 11.11 ± 8.04 (3) | 1.81 |
| SLC39A6* | 0.35 ± 0.12 (3) | 0.22 ± 0.14 (3) | 0.62 |  | SLC6A5* | 0.37 ± 0.14 (3) | 0.05 ± 0.02 (3) | 0.12 |
| SLC39A7 | 90.02 ± 42.61 (3) | 21.55 ± 4.88 (3) | 0.24 |  | SLC6A6 | 61.05 ± 23.04 (3) | 71.56 ± 20.61 (3) | 1.17 |
| SLC39A8 | 116.69 ± 74.83 (3) | 212.67 ± 36.92 (3) | 1.82 |  | SLC6A7 | 0.36 ± 0.08 (3) | 0.04 ± 0.02 (3) | 0.12 |
| SLC39A9 | 13.41 ± 13.31 (3) | 27.25 ± 3.67 (3) | 2.03 |  | SLC6A8* | 13.63 ± 7.56 (3) | 25.25 ± 12.59 (3) | 1.85 |
| SLC3A1 | 0.25 ± 0.09 (3) | 0.02 ± 0.01 (3) | 0.07 |  | SLC6A9 | 0.43 ± 0.24 (3) | 0.75 ± 1.2 (3) | 1.74 |
| SLC3A2 | 8.12 ± 3.48 (3) | 12.02 ± 1.93 (3) | 1.48 |  | SLC7A1 | 26.63 ± 4.71 (3) | 7.05 ± 2.28 (3) | 0.26 |
| SLC40A1 | 87.4 ± 13.87 (3) | 316.54 ± 163.59 (3) | 3.62 |  | SLC7A10 | 4.32 ± 1.72 (3) | 1.31 ± 0.88 (3) | 0.30 |
| SLC41A1 | 11.68 ± 7.17 (3) | 7.31 ± 9.42 (3) | 0.63 |  | SLC7A11* | 1.14 ± 0.59 (3) | 1.43 ± 1.22 (3) | 1.25 |
| SLC41A2 | 1.77 ± 1.11 (3) | 3.06 ± 3.44 (3) | 1.73 |  | SLC7A13 | 0.2 ± 0.1 (3) | 0.13 ± 0.19 (3) | 0.65 |
| SLC41A3 | 10.66 ± 4.23 (3) | 5.84 ± 1.75 (3) | 0.55 |  | SLC7A14 | 0.72 ± 0.2 (3) | 0.14 ± 0.04 (3) | 0.20 |
| SLC43A1 | 1.62 ± 1.42 (3) | 1.9 ± 0.26 (3) | 1.17 |  | SLC7A2 | 0.42 ± 0.04 (3) | 0.06 ± 0.06 (3) | 0.14 |
| SLC43A2 | 74.45 ± 38.33 (3) | 17.73 ± 4.81 (3) | 0.24 |  | SLC7A3 | 1.44 ± 1.84 (3) | 0.85 ± 1.18 (3) | 0.59 |
| SLC43A3 | 5.45 ± 2.81 (3) | 2.68 ± 0.71 (3) | 0.49 |  | SLC7A4 | 6.64 ± 2.58 (3) | 1.84 ± 1.19 (3) | 0.28 |
| SLC44A1 | 2.98 ± 0.43 (3) | 21.08 ± 15.98 (3) | 7.07 |  | SLC7A5 | 81.07 ± 30.72 (3) | 35.69 ± 5.6 (3) | 0.44 |
| SLC44A2 | 9.1 ± 4.56 (3) | 61.74 ± 42.94 (3) | 6.79 |  | SLC7A6 | 3.23 ± 2.86 (3) | 2.45 ± 2.4 (3) | 0.76 |
| SLC44A3 | 0.23 ± 0.1 (3) | 0.03 ± 0.01 (3) | 0.14 |  | SLC7A7 | 42.97 ± 14.75 (3) | 16.32 ± 4.85 (3) | 0.38 |
| SLC44A4* | 16.43 ± 3.74 (3) | 3.19 ± 1.25 (3) | 0.19 |  | SLC7A8 | 5.18 ± 4.62 (3) | 0.37 ± 0.28 (3) | 0.07 |
| SLC44A5 | 0.27 ± 0.15 (3) | 0.08 ± 0.05 (3) | 0.29 |  | SLC7A9 | 1.52 ± 1.06 (3) | 0.6 ± 0.22 (3) | 0.39 |
| SLC45A1 | 0.25 ± 0.14 (3) | 0.14 ± 0.15 (3) | 0.58 |  | SLC8A1* | 0.57 ± 0.13 (3) | 0.12 ± 0.05 (3) | 0.22 |
| SLC45A2 | 0.19 ± 0.12 (3) | 0.04 ± 0.02 (3) | 0.18 |  | SLC8A2 | 0.24 ± 0.16 (3) | 0.09 ± 0.08 (3) | 0.35 |
| SLC45A3 | 0.62 ± 0.4 (3) | 1.32 ± 0.89 (3) | 2.15 |  | SLC8A3 | 3.86 ± 1.35 (3) | 5.69 ± 3.81 (3) | 1.47 |
| SLC45A4 | 3.91 ± 3.66 (3) | 2.39 ± 1.41 (3) | 0.61 |  | SLC8B1 | 0.75 ± 0.43 (3) | 0.12 ± 0.08 (3) | 0.15 |
| SLC46A1 | 1.14 ± 1.08 (3) | 0.26 ± 0.27 (3) | 0.23 |  | SLC9A1* | 23.46 ± 7.58 (3) | 23.13 ± 13.27 (3) | 0.99 |
| SLC46A2 | 2.27 ± 1.9 (3) | 0.65 ± 0.47 (3) | 0.29 |  | SLC9A2 | 0.2 ± 0.07 (3) | 0.04 ± 0.01 (2) | - |
| SLC46A3 | 2.44 ± 1.14 (3) | 1.72 ± 1.36 (3) | 0.71 |  | SLC9A3* | 0.12 ± 0.03 (3) | 0.06 ± 0.04 (3) | 0.46 |
| SLC47A1 | 9.55 ± 12.18 (3) | 5.51 ± 4.13 (3) | 0.58 |  | SLC9A4 | 107.58 ± 36.21 (3) | 23.84 ± 12.48 (3) | 0.22 |
| SLC47A2 | 0.5 ± 0.26 (3) | 0.1 ± 0.05 (3) | 0.21 |  | SLC9A5 | 38.23 ± 20.7 (3) | 8.2 ± 4.81 (3) | 0.21 |
| SLC48A1 | 5.58 ± 3.13 (3) | 12.49 ± 2.45 (3) | 2.24 |  | SLC9A6 | 2.72 ± 2.82 (3) | 1.4 ± 1.18 (3) | 0.51 |
| SLC49A3 | 2.12 ± 2.72 (3) | 1.81 ± 0.61 (3) | 0.85 |  | SLC9A7 | 24.45 ± 4.63 (3) | 48.71 ± 16.01 (3) | 1.99 |
| SLC49A4 | 6.37 ± 1.13 (3) | 1.71 ± 0.48 (3) | 0.27 |  | SLC9A8 | 31.12 ± 19.03 (3) | 44.61 ± 7.82 (3) | 1.43 |
| SLC4A1 | 222.93 ± 70.54 (3) | 196.72 ± 79.63 (3) | 0.88 |  | SLC9A9 | 4.96 ± 3.91 (3) | 30.37 ± 12.65 (3) | 6.12 |
| SLC4A10 | 0.45 ± 0.17 (3) | 0.03 ± 0.02 (3) | 0.07 |  | SLC9B1 | 0.18 ± 0.07 (3) | 2.39 ± 0.7 (3) | 13.4 |
| SLC4A11 | 0.64 ± 0.76 (3) | 0.44 ± 0.16 (3) | 0.68 |  | SLC9B2 | 6.39 ± 3.65 (3) | 2.49 ± 1.23 (3) | 0.39 |
| SLC4A2 | 24.57 ± 8.32 (3) | 7.09 ± 0.53 (3) | 0.29 |  | SLC9C1 | 0.83 ± 0.63 (3) | 1.82 ± 1.29 (3) | 2.20 |
| SLC4A3 | 16.45 ± 15.26 (3) | 6.98 ± 6.27 (3) | 0.42 |  | SLCO1A2 | 1.09 ± 0.74 (3) | 0.12 ± 0.07 (3) | 0.11 |

**Table 2.** Continued.

The table lists data for 199 enzymes, 444 transporters, FcRn subunits and 11 transcription factors. Data are expressed as mean ± SD (n). FC, fold change relative to healthy baseline. FC was only calculated when the number of readouts in each sample set was n = 3 to allow meaningful average concentrations to be used. * Enzymes and transporters that are also drug targets.

| **Gene** | **Healthy** | **Cholestatic liver disease** | **FC** |
| --- | --- | --- | --- |
| SLCO1B1 | 0.08 ± 0.04 (3) | 0.04 ± 0.03 (3) | 0.55 |
| SLCO1B3 | 0.11 ± 0.11 (3) | 0.01 ± 0.01 (2) | - |
| SLCO1C1 | 0.41 ± 0.43 (3) | 0.11 ± 0.08 (3) | 0.26 |
| SLCO2A1 | 11.07 ± 7.10 (3) | 7.4 ± 4.06 (3) | 0.67 |
| SLCO2B1 | 2.52 ± 2.18 (3) | 0.45 ± 0.28 (3) | 0.18 |
| SLCO3A1 | 18.12 ± 19.77 (3) | 25.64 ± 12.83 (3) | 1.41 |
| SLCO4A1 | 20.87 ± 2.97 (3) | 3.82 ± 1.59 (3) | 0.18 |
| SLCO4C1 | 22.22 ± 7.9 (3) | 8.89 ± 4.81 (3) | 0.40 |
| SLCO5A1 | 0.6 ± 0.25 (3) | 0.32 ± 0.43 (3) | 0.54 |
| SLCO6A1 | 0.13 ± 0.04 (3) | 0.02 ± 0.02 (3) | 0.17 |
| SULT1A1 | 167.27 ± 65.54 (3) | 21.86 ± 6.5 (3) | 0.13 |
| SULT1A2 | 22.29 ± 8.1 (3) | 4.4 ± 2.33 (3) | 0.20 |
| SULT1A4 | 0.78 ± 0.84 (3) | 0.36 ± 0.25 (3) | 0.46 |
| SULT1B1 | 328.09 ± 37.51 (3) | 127.08 ± 20.08 (3) | 0.39 |
| SULT1C2 | 3.79 ± 2.72 (3) | 1.57 ± 1.74 (3) | 0.41 |
| SULT1C3 | 0.16 ± 0.05 (3) | 0.02 ± 0.01 (3) | 0.12 |
| SULT1C4 | 1.69 ± 1.35 (3) | 2.18 ± 0.41 (2) | - |
| SULT1E1* | 0.04 ± 0.04 (3) | 0.01 ± 0.01 (2) | - |
| SULT2A1 | 0.25 ± 0.02 (3) | 1.09 ± 0.92 (3) | 4.31 |
| SULT2B1 | 0.35 ± 0.04 (3) | 0.08 ± 0.03 (3) | 0.23 |
| SULT4A1 | 0.34 ± 0.09 (3) | 0.09 ± 0.02 (3) | 0.26 |
| SULT6B1 | 0.16 ± 0.14 (3) | 0.02 (1) | - |
| TAP1 | 21.7 ± 8.49 (3) | 14.81 ± 2.23 (3) | 0.68 |
| TAP2 | 7.49 ± 4.41 (3) | 8.1 ± 3.66 (3) | 1.08 |
| TPMT* | 16.5 ± 8.69 (3) | 8.7 ± 1.2 (3) | 0.53 |
| UGT1A1 | 0.25 ± 0.14 (3) | 0.02 ± 0.02 (2) | - |
| UGT1A10 | 98.45 ± 7.71 (3) | 15.41 ± 4.08 (3) | 0.16 |
| UGT1A3 | 0.04 ± 0.03 (3) | - | - |
| UGT1A4 | 0.02 ± 0.02 (2) | 0.01 (1) | - |
| UGT1A6 | 0.07 ± 0.01 (3) | 0.02 ± 0.002 (3) | 0.25 |
| UGT1A7 | 75.15 ± 17.43 (3) | 13.73 ± 7.35 (3) | 0.18 |
| UGT1A8 | 53.55 ± 23.44 (3) | 10.19 ± 4.52 (3) | 0.19 |
| UGT1A9 | 44.13 ± 14.03 (3) | 7.79 ± 2.74 (3) | 0.18 |
| UGT2A1 | 0.04 ± 0.03 (3) | 0.02 ± 0.01 (3) | 0.57 |
| UGT2A3 | 1.13 ± 0.72 (3) | 0.33 ± 0.27 (3) | 0.29 |
| UGT2B10 | 0.14 ± 0.11 (3) | 0.17 ± 0.15 (3) | 1.23 |
| UGT2B11 | 0.19 ± 0.11 (3) | 0.55 ± 0.06 (3) | 2.84 |
| UGT2B15 | 0.09 ± 0.04 (3) | 0.14 ± 0.16 (2) | - |
| UGT2B17 | 0.09 ± 0.07 (3) | 1.21 ± 1.33 (2) | - |
| UGT2B28 | 0.05 ± 0.03 (3) | 0.02 ± 0.002 (2) | - |
| UGT2B4 | 0.98 ± 0.38 (3) | 0.67 ± 0.58 (3) | 0.68 |
| UGT2B7 | 0.15 ± 0.16 (3) | 0.64 ± 0.39 (3) | 4.29 |
| UGT3A1 | 0.32 ± 0.03 (3) | 0.04 ± 0.02 (2) | - |
| UGT3A2 | 1.3 ± 0.52 (3) | 0.26 ± 0.28 (3) | 0.20 |
| XDH | 0.65 ± 0.14 (3) | 0.05 ± 0.01 (3) | 0.08 |
| NR1H4* | 0.17 ± 0.09 (3) | 0.33 ± 0.56 (3) | 2.01 |
| NR1H3 | 4.21 ± 2.09 (3) | 4.92 ± 3.28 (3) | 1.17 |
| NR1I2 | 21.11 ± 6.2 (3) | 3.32 ± 1.07 (3) | 0.16 |
| NR1I3 | 0.72 ± 0.24 (3) | 0.5 ± 0.28 (3) | 0.69 |
| PPARA* | 25.49 ± 13.43 (3) | 23.8 ± 11.03 (3) | 0.93 |
| PPARD* | 15.94 ± 8.63 (3) | 9.84 ± 7.3 (3) | 0.62 |
| PPARG* | 55.95 ± 61.66 (3) | 50.63 ± 54.22 (3) | 0.90 |
| RXRA | 73.27 ± 26.82 (3) | 41.14 ± 12.02 (3) | 0.56 |
| RXRB | 10.69 ± 1.64 (3) | 9.07 ± 4.5 (3) | 0.85 |
| RXRG | 1.16 ± 0.88 (3) | 0.08 ± 0.08 (3) | 0.06 |
| VDR* | 14.16 ± 12.61 (3) | 12.78 ± 7.73 (3) | 0.90 |

**Table S3.** Cholestatic liver disease population model parameters.

| **Base Simcyp model** | Sim-Healthy Volunteers (Simcyp library) | | | |
| --- | --- | --- | --- | --- |
| **Parameter [units]** | **FC** | **Modified mean** | **CV** | **References** |
| CYP2C9 [pmol/mg protein] | 0.55 | 42.74 | 64% | Present study, Simcyp library |
| CYP2C19 [pmol/mg protein] | 0.79 | 3.48 | 52% | Present study, Simcyp library |
| CYP3A4 [pmol/mg protein] | 1.06 | 145.22 | 41% | Present study, Simcyp library |
| CYP3A5 [pmol/mg protein] | 4.25 | 437.75 | 65% | Present study, Simcyp library |
| CYP2D6 [pmol/mg protein] | 0.37 | 3.48 | 65% | Present study, Simcyp library |
| UGT1A6 [pmol/mg protein] | 0.25 | 5.00 | 30% | Present study, Simcyp library |
| UGT2B4 [pmol/mg protein] | 0.68 | 36.72 | 28% | Present study, Simcyp library |
| UGT2B7 [pmol/mg protein] | 4.29 | 304.59 | 30.4% | Present study, Simcyp library |
| OATP1B1 (SLCO1B1) [pmol/10^6^ hepatocytes] | 0.55 | 1.71 | 73% | Present study, Simcyp library |
| MRP2 (ABCC2) [pmol/10^6^ hepatocytes] | 0.17 | 0.10 | 88% | Present study, Simcyp library |
| MRP3 (ABCC3) [pmol/10^6^ hepatocytes] | 12.51 | 2.99 | 65% | Present study, Simcyp library |
| P-gp (ABCB1) [pmol/10^6^ hepatocytes] | 0.19 | 0.05 | 59% | Present study, Simcyp library |
| BCRP (ABCG2) [pmol/10^6^ hepatocytes] | 0.58 | 0.06 | 30% | Present study, Simcyp library |
| BSEP (ABCB11) [pmol/10^6^ hepatocytes] | 0.42 | 0.42 | 131% | Present study, Simcyp library |
| HSA [g/L] | - | 40 | 10% | [1] |
| Bilirubin production rate [mg/kg/day] | - | 3.8 | 16% | [2] |
| Trial design: demographics | 9:1 female:male ratio; age 20-60 years | | | [1] |

The model was developed to simulate drug exposure in primary biliary cholangitis (PBC). FC, fold change based on RNAseq data; CV, coefficient of variation calculated as (%CV = 100 * SD / Mean); HSA, human serum albumin; Variability (CV) was based on default model variability, except UGT1A1 which was changed from 24% to a typical default of 30%.

**References**

[1] M. Alomari, F. Covut, L. Al Momani, P. Chadalavada, A. Hitawala, M.F. Young, C. Romero-Marrero, Evaluation of the United Kingdom-primary biliary cholangitis and global primary biliary cholangitis group prognostic models for primary biliary cholangitis patients treated with ursodeoxycholic acid in the U.S. population, JGH Open 4 (2019) 132–139.

[2] P.D. Berk, R.B. Howe, J.R. Bloomer, N.I. Berlin, Studies of bilirubin kinetics in normal adults, J. Clin. Invest. 48 (1969) 2176–2190.
